# Lyssaviruses in Insectivorous Bats, South Africa, 2003–2018

**DOI:** 10.3201/eid2612.203592

**Published:** 2020-12

**Authors:** Jessica Coertse, Colyn S. Grobler, Claude T. Sabeta, Ernest C.J. Seamark, Teresa Kearney, Janusz T. Paweska, Wanda Markotter

**Affiliations:** University of Pretoria, Pretoria, South Africa (J. Coertse, C.S. Grobler, T. Kearney, J.T. Paweska, W. Markotter);; National Institute of Communicable Diseases of the National Health Laboratory Service, Sandringham, South Africa (J. Coertse, J.T. Paweska);; Onderstepoort Veterinary Institute, Pretoria (C.T. Sabeta);; AfricanBats NPC, Pretoria (E.C.J. Seamark, T. Kearney);; Ditsong National Museum of Natural History, Pretoria (T. Kearney)

**Keywords:** rabies, lyssaviruses, South Africa, bats, Duvenhage virus, Matlo bat lyssavirus, surveillance, zoonoses, viruses, encephalitis, insectivorous bats, West Caucasian bat virus, viral zoonoses

## Abstract

We detected 3 lyssaviruses in insectivorous bats sampled in South Africa during 2003–2018. We used phylogenetic analysis to identify Duvenhage lyssavirus and a potentially new lyssavirus, provisionally named Matlo bat lyssavirus, that is related to West Caucasian bat virus. These new detections highlight that much about lyssaviruses remains unknown.

Lyssaviruses cause fatal encephalitic disease in mammals; 6 viral species have been implicated in human deaths (*1*). Bats are the primary hosts for members of the *Lyssavirus* genus, which belongs to the family *Rhabdoviridae*. Researchers have described 17 lyssavirus species, and a putative species is awaiting formal classification (*1*). The genus can be divided into >3 phylogroups on the basis of genetic, immunogenic, and pathogenic properties (*2*). Rabies vaccines and postexposure prophylaxis protect against infection by members of phylogroup I but provide limited protection against phylogroups II or III (*3*). 

In Africa, 6 lyssaviruses are in circulation: rabies virus, which is associated with terrestrial carnivores; Duvenhage virus (DUVV), which is associated with insectivorous bats, specifically the Egyptian slit-faced bat (*Nycteris thebaica*); Lagos bat virus, which is associated with various species of frugivorous bats; Mokola virus, for which the reservoir host is unknown; Shimoni bat virus, which is associated with the striped leaf-nosed bat (*Macronycteris vittatus*); and Ikoma lyssavirus, for which the reservoir host is unknown (*1*). Only rabies virus, DUVV, and Mokola virus have been associated with human deaths on the continent. Lyssavirus surveillance in Africa is inadequate. As a result, genetic diversity, geographic distribution, and host species associations of lyssaviruses are poorly understood (*1*). However, this information is crucial for making treatment decisions, especially in resource-limited settings (*1*).

We report the results of 16 years of surveillance of insectivorous bats in South Africa. We used genetic characterization to identify DUVV and a potential novel lyssavirus from phylogroup III.

## The Study

**Figure 1:**
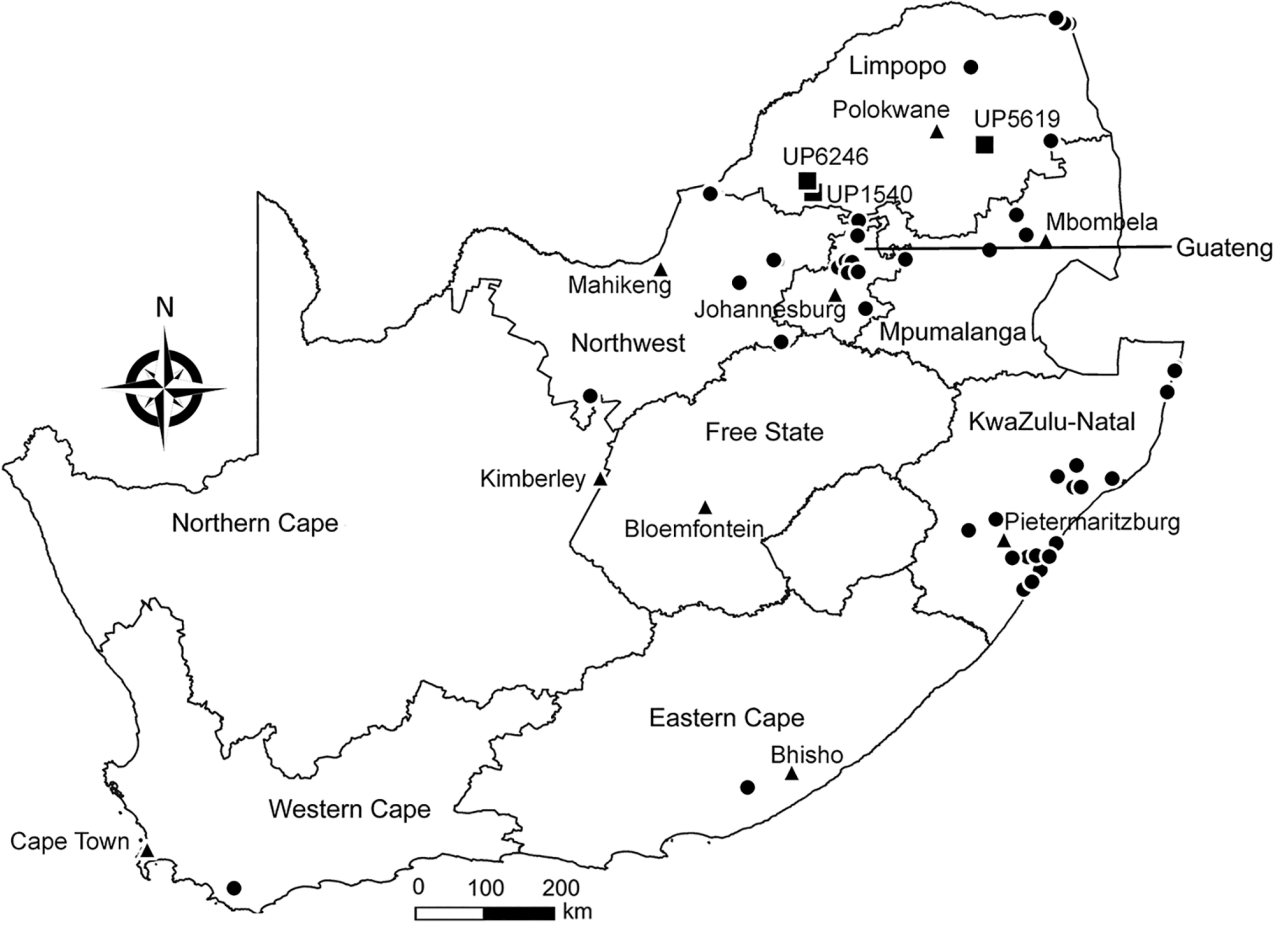
Locations of bat collection sites, South Africa, 2003–2018. Circles indicate collection sites, squares indicate sites with lyssavirus-positive bats, and triangles indicate capitals.

During 2003–2018, we tested 605 insectivorous bats (Appendix Tables 1, 2) of 41 species across South Africa (Figure 1; Appendix Tables 1, 2). Most bats were collected as part of a broader biosurveillance program in collaboration with bat taxonomists for species identification and classification. Among the bats collected, 562 appeared healthy and 28 were dead. Another 12 exhibited signs of disease or abnormal behavior, and 3 had been involved in human contact; we submitted these samples for rabies testing.

We anesthetized the sampled bats with isoflurane inhalant (Safeline Pharmaceuticals, https://safeline.co.za), exsanguinated them by cardiac puncture, and then performed full necropsies. We identified bats on the basis of morphologic (*4*) and genetic characteristics (*5*). The sampling protocol was approved by the University of Pretoria Animal Ethics Committee (approval no. EC054-14). Permission to conduct research was obtained from the Department of Agriculture, Land Reform and Rural Development (formerly Department of Agriculture, Forestry, and Fisheries) of the Republic of South Africa under Section 20 of the Animal Diseases Act 1984 with additional provincial permits granted (Appendix Table 3).

We extracted total RNA from the bats’ brain material and subjected it to real-time reverse transcription PCR selective for 126 bp of the nucleoprotein gene (*6*). Samples from 3 bats tested positive for lyssavirus RNA; we sequenced the amplicons (*7*). One of these samples came from an Egyptian slit-faced bat (sample no. UP1540) collected in 2012 from the Rooiberg area, Limpopo. This bat, which was collected by members of a bat interest group in August 2012, tested positive for DUVV (Figure 1). We also obtained sequences of a potentially novel lyssavirus in samples from 2 apparently healthy Natal long-fingered bats (*Miniopterus natalensis*, sample nos. UP5619 and UP6246); these sequences were distantly related to West Caucasian bat virus (WCBV). We collected these samples from Matlapitsi cave, Limpopo, in 2015 and Madimatle cave, Limpopo, in 2016 (Figure 1).

To infer the phylogeny, we used the complete nucleoprotein gene sequences of the 3 lyssaviruses according to a method described previously (*8*). We used Bayesian inference to compare our samples with representative sequences from GenBank (Appendix Table 4). We analyzed the dataset with BEAST (*9*); we used the general time-reversible substitution model as determined by jmodeltest2 (*10*), invariant sites, and gamma distribution. We assumed an underlying coalescent process with constant population size and Markov chain Monte Carlo chains of 50 million generations.

**Figure 2:**
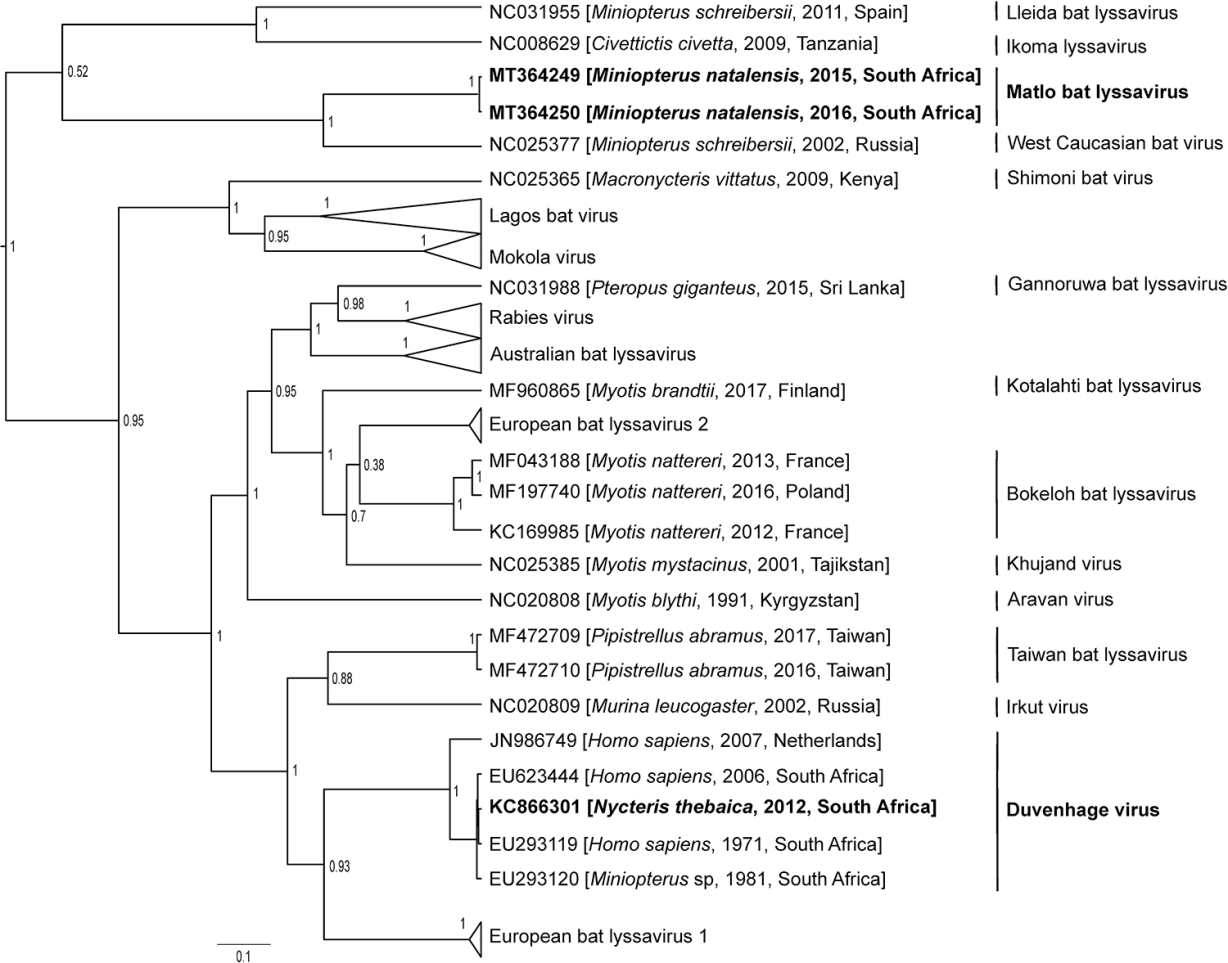
Phylogenetic reconstruction by Bayesian inference of nucleoprotein gene sequences of lyssavirus sequences from bats collected in South Africa, 2003–2018 (bold), and other representative lyssaviruses. Node numbers indicate posterior probabilities. GenBank accession number, host species, year of detection, and country of origin are indicated for each sequence. Scale bar indicates number of substitutions per site.

The results indicated that the sequences from Natal long-fingered bats were most closely related to, but distinct from, WCBV (Figure 2); they might belong to a novel species of *Lyssavirus*, provisionally called Matlo bat lyssavirus (MBLV). These 2 novel sequences had a shared nucleotide identity of 99.2%. Compared with other lyssaviruses, they shared the highest nucleotide identity with WCBV (80.9%–81%) and the lowest with Ikoma lyssavirus (70.5%). The DUVV sequence was 91.6%–99.3%, similar to previously described DUVV sequences (Table).

**Table:** Genetic similarities of 3 lyssaviruses found in insectivorous bats in South Africa, 2003–2018, and representative lyssavirus species from GenBank*

Virus	% Similarity

UP5619 (MT364249)			UP6246 (MT364250)			UP1540 (KC866301)	
Nucleotide	Amino acid	Nucleotide	Amino acid	Nucleotide	Amino acid
Aravan virus	72.7	83.3		72.6	84.0		NA	NA
Australian bat lyssavirus	72.7–74.4	82–83.3		72.8–74.7	82.4–83.7		NA	NA
Bokeloh bat lyssavirus	72.9–73.3	81.5		73–73.4	81.8		NA	NA
European bat lyssavirus 1	71.1–72.4	82.2–82.4		71.2–72.5	82.7–82.9		NA	NA
European bat lyssavirus 2	71–72.4	80.0–80.7		71–72.4	80.2–80.9		NA	NA
Gannoruwa bat lyssavirus	70.6	83.1		70.6	83.5		NA	NA
Ikoma lyssavirus	70.5	79.5		70.5	79.5		NA	NA
Irkut virus	72	83.3		72.4	83.8		NA	NA
Khujand virus	72.5	81.3		72.5	81.8		NA	NA
Kotalahti bat lyssavirus	71.9	81.8		71.9	82.4		NA	NA
Lagos bat virus	71.1–74.2	82.6–84		71.3–74.5	83.1–84.4		NA	NA
Lleida bat lyssavirus	71.3	81.5		71.4	81.5		NA	NA
Mokola virus	72.2–73.8	82.4–83.7		72.3–73.7	82.8–84.2		NA	NA
Rabies virus	71.1–72.4	80.6–82		71–72.2	80.8–82.4		NA	NA
Shimoni bat virus	73.1	83.5		73.6	84.2		NA	NA
Taiwan bat lyssavirus	73.6–73.9	83.3		73.7–73.9	83.8		NA	NA
West Caucasian bat virus	80.9	95.5		81	96.2		NA	NA
DUVVs								
EU293119	72.9	84		72.9	84.4		99.3	99.5
EU293120	72.9	84		72.9	84.4		98.5	99.5
JN986749	73	84		73	84.4		91.6	99.3
EU623444	72.8	84		72.8	84.4		98.6	99.3

*GenBank accession numbers are shown for sequences from this study and reference DUVV sequences. DUVV, Duvenhage virus; NA, not applicable.

## Conclusions

From 2003–2018, we detected 3 lyssavirus infections in insectivorous bats from South Africa, indicating active but low-level circulation of lyssaviruses in this population. The Egyptian slit-faced bat is the only species of bat that has been conclusively linked with DUVV (*11*). Our finding is 1 of only 6 known DUVV cases, 3 of which caused fatal infection in humans (*1*). This finding suggests that these infections are underreported. The Egyptian slit-faced bat is widely distributed in Africa. It co-roosts with bats of various other species and switches roosts frequently, implicating a wider potential to infect other species (*12*).

We detected a novel lyssavirus, MBLV, that belongs to phylogroup III and is most closely related to a sequence of WCBV that was isolated from a common long-fingered bat (*Miniopterus schreibersii*) from the Russian Caucasus in 2002 (*13*). The nucleotide identity for MBLV falls within the species demarcation criteria determined by the International Committee for the Taxonomy of Viruses of 80%–82% for the complete nucleoprotein gene (*2*). If MBLV is not pronounced a new lyssavirus species, this virus would be a distinct lineage of WCBV. In 2006–2007, detection of virus neutralizing antibodies (seroprevalence 17%–26%) against WCBV in *Miniopterus* bats in Kenya spurred speculation that WCBV, or a closely related virus, was circulating in Africa (*14*). The bat family Miniopteridae, which includes >23 species in Africa, does not host known lyssaviruses in phylogroup I or II (*12*). The long-fingered bats are widely distributed throughout Africa (*12*).

Models have demonstrated that WCBV can cause fatal encephalitis in animals; commercial human and veterinary vaccines do not offer significant protection (*3*). Considering genetic diversity and phylogenetic grouping, we speculate that current vaccines will probably provide little to no protection against infection with MBLV (as with WCBV). Because of the lack of diagnostic capability in Africa (*6*), the potential threat of this virus is unknown. Continued surveillance and development of improved pharmaceuticals are necessary for the prevention of these infections. We observed a low prevalence (0.5%), similar to other lyssaviruses (*1*). Additional longitudinal surveillance, including serologic testing, among bats of this species and other potential hosts must be implemented to determine if Natal long-fingered bats are the reservoir host of MBLV. This study did not obtain equally representative samples of all bat species from all sampling sites; MBLV might exist in other bat species in South Africa. 

In summary, the mechanisms of lyssavirus maintenance in bats is still unknown and could be influenced by various environmental and ecologic factors (*1*). Additional surveillance and comparative seroprevalence studies are needed to establish the host range and distribution of MBLV and other lyssaviruses. Although the public health impact of MBLV is currently unknown, DUVV can cause fatal infection and should be taken seriously. Surveillance is needed to understand the epidemiology and diversity of bat lyssaviruses and inform prevention efforts. 

Appendix Additional information on lyssaviruses in insectivorous bats, South Africa, 2003–2018.
