# Supplementary material for: Lyssaviruses in Insectivorous Bats, South Africa, 2003–2018
Source: Emerg Infect Dis. 2020 Dec;26(12):3056–60. doi: 10.3201/eid2612.203592 (PMC7706942; doi:10.3201/eid2612.203592)
Supplement: Appendix — Additional information on lyssaviruses in insectivorous bats, South Africa, 2003–2018. [file 20-3592-Techapp-s1.pdf]

Article DOI: https://doi.org/10.3201/eid2612.203592

Lyssaviruses in Insectivorous Bats, South Africa, 2003–2018

Appendix

Appendix Table 1. Bats collected for lyssavirus surveillance, South Africa, 2003–2018

Laboratory no.*	Collection date	Bat species†	ID method	Province	Coordinates	SIte
UP 0004	2004 Aug 20	*Neoromicia (Laephotis) nana*	Morphologic	Mpumalanga	-34.472989, 19.668606	Laughing Waters Farm, Nelspruit
UP 0005	2004 Aug 21	*Neoromicia (Laephotis) nana*	Morphologic	Mpumalanga	-34.472989, 19.668606	Laughing Waters Farm, Nelspruit
UP 0006	2004 Aug 21	*Neoromicia (Laephotis) nana*	Morphologic	Mpumalanga	-34.472989, 19.668606	Laughing Waters Farm, Nelspruit
UP 0012	2004 Aug 26	*Pipistrellus hesperidus*	Genetic	Eastern Cape	-33.069128, 26.816924	Andries Vosloo Kudu Reserve, Grahamstown
UP 0013	2004 Aug 26	*Pipistrellus hesperidus*	Genetic	Eastern Cape	-33.069128, 26.816924	Andries Vosloo Kudu Reserve, Grahamstown
UP 0014	2004 Aug 26	*Pipistrellus hesperidus*	Genetic	Eastern Cape	-33.069128, 26.816924	Andries Vosloo Kudu Reserve, Grahamstown
UP 0015	2004 Aug 26	*Pipistrellus hesperidus*	Genetic	Eastern Cape	-33.069128, 26.816924	Andries Vosloo Kudu Reserve, Grahamstown
UP 0016	2004 Aug 26	*Pipistrellus hesperidus*	Genetic	Eastern Cape	-33.069128, 26.816924	Andries Vosloo Kudu Reserve, Grahamstown
UP 0017	2004 Aug 26	*Pipistrellus hesperidus*	Genetic	Eastern Cape	-33.069128, 26.816924	Andries Vosloo Kudu Reserve, Grahamstown
UP 0018	2004 Aug 26	*Pipistrellus hesperidus*	Genetic	Eastern Cape	-33.069128, 26.816924	Andries Vosloo Kudu Reserve, Grahamstown
UP 0056, DM7842	2004 May 02	*Nycteris thebaica*	Museum voucher	KwaZulu-Natal	–29.857855, 30.722425	Shongeni Valley, Shongweni Nature Reserve
UP 0057, DM8002	2003 Feb 20	*Otomops martiensseni*	Museum voucher	KwaZulu-Natal	–29.851041, 31.021642	Durban
UP 0058	2004 Apr 15	*Pipistrellus hesperidus*	Morphologic	KwaZulu-Natal	–28.735800, 31.136096	Nkandla Forest Reserve, Nkandla
UP 0059, DM7838	2003 Nov 11	*Nycteris thebaica*	Museum voucher	KwaZulu-Natal	–29.834418, 30.926404	Westville
UP 0060	2004 Apr 15	*Pipistrellus hesperidus*	Morphologic	KwaZulu-Natal	–28.735800, 31.136096	Nkandla Forest Reserve, Nkandla
UP 0061, DM7906	2003 May 26	*Scotophilus dinganii*	Museum voucher	KwaZulu-Natal	Not recorded	Not recorded
UP 0063	2004 Jun 17	*Pipistrellus hesperidus*	Morphologic	KwaZulu-Natal	-30.036554, 30.896156	Amanzimtoti
UP 0064, DM8013	2005 Feb 23	*Pipistrellus hesperidus*	Museum voucher	KwaZulu-Natal	–27.559892, 32.664073	Sodwana Bay National Park
UP 0065	2004 May 15	*Rhinolophus darlingi*	Morphologic	KwaZulu-Natal	-30.310606, 30.664202	Umzino
UP 0066, DM7910	2003 Apr 22	*Chaerephon (Mops) pumilus*	Museum voucher	KwaZulu-Natal	–29.839963, 30.840120	Pinetown
UP 0067, DM7913	2002 Sep 01	*Chaerephon (Mops) pumilus*	Museum voucher	KwaZulu-Natal	-30.115940, 30.837709	Illovo
UP 0068, DM8004	2004 May 19	*Scotophilus dinganii*	Museum voucher	KwaZulu-Natal	–29.851041, 31.021642	Durban
UP 0070, DM7914	2004 May 27	*Otomops martiensseni*	Museum voucher	KwaZulu-Natal	–29.851041, 31.021642	Durban
UP 0071, DM8382	2005 May 15	*Miniopterus natalensis*	Museum voucher	KwaZulu-Natal	–28.582087, 31.401064	Melmoth
UP 0082, DM8376	2005 May 15	*Rhinolophus clivosus*	Museum voucher	KwaZulu-Natal	–28.582087, 31.401064	Melmoth
UP 0084, DM8373	2005 May 14	*Rhinolophus clivosus*	Museum voucher	KwaZulu-Natal	–28.882841, 31.464582	Eshowe

Laboratory no.*	Collection date	Bat species†	ID method	Province	Coordinates	SIte
UP 0085, DM7848	2005 Jun 24	*Scotophilus dinganii*	Museum voucher	KwaZulu-Natal	–29.851041, 31.021642	Durban
UP 0098, DM8439	2005 Aug 08	*Chaerephon (Mops) pumilus*	Museum voucher	KwaZulu-Natal	–28.764711, 31.897268	Empangeni
UP 0099, DM8381	2005 May 15	*Miniopterus natalensis*	Museum voucher	KwaZulu-Natal	–28.582087, 31.401064	Melmoth
UP 0100, DM8383	2005 May 15	*Miniopterus natalensis*	Museum voucher	KwaZulu-Natal	–28.582087, 31.401064	Melmoth
UP 0101, DM7915	2005 Jun 24	*Neoromicia (Laephotis) nana*	Museum voucher	KwaZulu-Natal	–29.851041, 31.021642	Durban
UP 0102, DM8379	2005 May 15	*Rhinolophus clivosus*	Museum voucher	KwaZulu-Natal	–28.582087, 31.401064	Melmoth
UP 0103, DM8384	2005 May 15	*Miniopterus natalensis*	Museum voucher	KwaZulu-Natal	–28.582087, 31.401064	Melmoth
UP 0104	2004 Sep 28	*Rhinolophus hildebrandtii* s.l.	Morphologic	Mpumalanga	Not recorded	Not recorded
UP 0109, DM7886	2004 Sep 28	*Rhinolophus hildebrandtii* s.l.	Museum voucher	Mpumalanga	Not recorded	Not recorded
UP 0111, DM7909	2004 May 17	*Otomops martiensseni*	Museum voucher	KwaZulu-Natal	–29.851041, 31.021642	Durban
UP 0115, DM8369	2005 May 14	*Miniopterus natalensis*	Museum voucher	KwaZulu-Natal	–28.885552, 31.361206	Entumeni
UP 0116, DM7908	2003 Feb 07	*Nycteris thebaica*	Museum voucher	KwaZulu-Natal	–29.851041, 31.021642	Durban
UP 0118, DM8374	2005 May 14	*Rhinolophus clivosus*	Museum voucher	KwaZulu-Natal	–29.851041, 31.021642	Durban
UP 0119, DM8001	2003 Feb 07	*Chaerephon (Mops) pumilus*	Museum voucher	KwaZulu-Natal	–29.851041, 31.021642	Durban
UP 0124, DM8000	2003 Apr 25	*Neoromicia (Laephotis) nana*	Museum voucher	KwaZulu-Natal	–29.668920, 31.118235	Umdloti
UP 0125, DM8380	2005 May 15	*Miniopterus natalensis*	Museum voucher	KwaZulu-Natal	–28.582087, 31.401064	Melmoth
UP 0126, DM8003	2004 Feb 27	*Chaerephon (Mops) pumilus*	Museum voucher	KwaZulu-Natal	-30.036554, 30.896156	Amanzimtoti
UP 0127, DM7904	2002 Dec 17	*Miniopterus natalensis*	Museum voucher	KwaZulu-Natal	–29.870801, 30.506680	Eston
UP 0129, DM8377	2005 May 15	*Rhinolophus clivosus*	Museum voucher	KwaZulu-Natal	–28.582087, 31.401064	Melmoth
UP 0131, DM8375	2005 May 14	*Rhinolophus clivosus*	Museum voucher	KwaZulu-Natal	–28.882841, 31.464582	Eshowe
UP 0132, DM8440	2006 Jan 25	*Chaerephon (Mops) pumilus*	Museum voucher	KwaZulu-Natal	–29.851041, 31.021642	Durban
UP 0134, DM7907	2004 Jan 04	*Chaerephon (Mops) pumilus*	Museum voucher	KwaZulu-Natal	Not recorded	Not recorded
UP 0135, DM8421	2005 Sep 01	*Otomops martiensseni*	Museum voucher	KwaZulu-Natal	–29.839963, 30.840120	Pinetown
UP 0138, DM8378	2005 May 15	*Rhinolophus clivosus*	Museum voucher	KwaZulu-Natal	–28.582087, 31.401064	Melmoth
UP 0139, DM8429	2005 Aug 01	*Miniopterus natalensis*	Museum voucher	KwaZulu-Natal	Not recorded	Not recorded
UP 0140, DM8419	2006 Jan 25	*Otomops martiensseni*	Museum voucher	KwaZulu-Natal	Not recorded	Not recorded
UP 0141, DM8420	2006 Jan 25	*Otomops martiensseni*	Museum voucher	KwaZulu-Natal	–29.851041, 31.021642	Durban
UP 0148	2006 Jun 23	*Otomops martiensseni*	Morphologic	KwaZulu-Natal	–29.851041, 31.021642	Durban
UP 0149	2006 May 29	*Chaerephon (Mops)pumilus*	Genetic	KwaZulu-Natal	Not recorded	Not recorded
UP 0151	2006 Mar 25	*Scotophilus dinganii*	Genetic	KwaZulu-Natal	–29.851041, 31.021642	Durban
UP 0152	2006 Apr 20	*Tadarida aegyptiaca*	Genetic	KwaZulu-Natal	–29.851041, 31.021642	Durban
UP 0153	2006 Apr 10	*Chaerephon (Mops) pumilus*	Genetic	KwaZulu-Natal	–29.851041, 31.021642	Durban
UP 0154, TM47967	2007 Jun 10	*Myotis welwitschii*	Museum voucher	Mpumalanga	–25.580473, 30.190405	Vlakfontein
UP 0155, TM47871	2006 Jan 11	*Myotis tricolor*	Museum voucher	Mpumalanga	–25.369997, 30.699825	Sudwala kraal

Laboratory no.*	Collection date	Bat species†	ID method	Province	Coordinates	SIte
UP 0156, TM48048	2007 Dec 16	*Tadarida aegyptiaca*	Museum voucher	North West	–27.6245, 24.63344	Taung World Heritage Site
UP 0157, TM48049	2007 Dec 16	*Tadarida aegyptiaca*	Museum voucher	North West	–27.6245, 24.63344	Taung World Heritage Site
UP 0158, TM48050	2007 Dec 16	*Tadarida aegyptiaca*	Museum voucher	North West	–27.6245, 24.63344	Taung World Heritage Site
UP 0159, TM48047	2007 Dec 16	*Tadarida aegyptiaca*	Museum voucher	North West	–27.6245, 24.63344	Taung World Heritage Site
UP 0160, TM48034	2007 Dec 14	*Rhinolophus denti*	Museum voucher	North West	–27.61543, 24.63005	Taung World Heritage Site
UP 0161, TM48032	2007 Dec 14	*Neoromicia (Laephotis) capensis*	Museum voucher	North West	–27.61543, 24.63005	Taung World Heritage Site
UP 0162, TM48036	2007 Dec 14	*Rhinolophus denti*	Museum voucher	North West	–27.61543, 24.63005	Taung World Heritage Site
UP 0163, TM48035	2007 Dec 14	*Rhinolophus denti*	Museum voucher	North West	–27.61543, 24.63005	Taung World Heritage Site
UP 0164, TM48033	2007 Dec 14	*Neoromicia (Laephotis) capensis*	Museum voucher	North West	–27.61543, 24.63005	Taung World Heritage Site
UP 0165, TM48042	2007 Dec 15	*Neoromicia (Laephotis) capensis*	Museum voucher	North West	–27.61376, 24.62976	Taung World Heritage Site
UP 0166, TM48046	2007 Dec 15	*Neoromicia (Laephotis) capensis*	Genetic, Museum voucher	North West	–27.61428, 24.62896	Taung World Heritage Site
UP 0167, TM48043	2007 Dec 15	*Neoromicia (Laephotis) capensis*	Museum voucher	North West	–27.61428, 24.62896	Taung World Heritage Site
UP 0168, TM48044	2007 Dec 15	*Neoromicia (Laephotis) capensis*	Museum voucher	North West	–27.61428, 24.62896	Taung World Heritage Site
UP 0169, TM48045	2007 Dec 15	*Neoromicia (Laephotis) capensis*	Museum voucher	North West	–27.61428, 24.62896	Taung World Heritage Site
UP 0171, TM48037	2007 Dec 14	*Rhinolophus denti*	Museum voucher	North West	–27.61543, 24.63005	Taung World Heritage Site
UP 0172, TM48041	2007 Dec 15	*Rhinolophus denti*	Museum voucher	North West	–27.61408, 24.62941	Taung World Heritage Site
UP 0173, TM48038	2007 Dec 15	*Eptesicus hottentotus*	Museum voucher	North West	–27.61408, 24.62941	Taung World Heritage Site
UP 0174, TM48040	2007 Dec 15	*Rhinolophus damarensis*	Museum voucher	North West	–27.61408, 24.62941	Taung World Heritage Site
UP 0190, TM48014	2007 Dec 01	*Nycteris thebaica*	Museum voucher	North West	–24.76622, 26.37098	Madikwe Game Reserve
UP 0192, TM48016	2007 Dec 01	*Rhinolophus simulator*	Museum voucher	North West	–24.76622, 26.37098	Madikwe Game Reserve
UP 0193, TM48017	2007 Dec 01	*Rhinolophus simulator*	Museum voucher	North West	–24.76622, 26.37098	Madikwe Game Reserve
UP 0194, TM48018	2007 Dec 01	*Rhinolophus damarensis*	Museum voucher	North West	–24.76622, 26.37098	Madikwe Game Reserve
UP 0195, TM48019	2007 Dec 01	*Rhinolophus damarensis*	Museum voucher	North West	–24.76622, 26.37098	Madikwe Game Reserve
UP 0196, TM48020	2007 Dec 01	*Rhinolophus damarensis*	Museum voucher	North West	–24.76622, 26.37098	Madikwe Game Reserve
UP 0197, TM48021	2007 Dec 01	*Rhinolophus damarensis*	Museum voucher	North West	–24.76622, 26.37098	Madikwe Game Reserve
UP 0198, TM48022	2007 Dec 01	*Miniopterus natalensis*	Museum voucher	North West	–24.76622, 26.37098	Madikwe Game Reserve
UP 0199, TM48023	2007 Dec 01	*Miniopterus natalensis*	Museum voucher	North West	–24.76622, 26.37098	Madikwe Game Reserve
UP 0200, TM48024	2007 Dec 01	*Miniopterus natalensis*	Museum voucher	North West	–24.76622, 26.37098	Madikwe Game Reserve
UP 0201, TM48025	2007 Dec 01	*Miniopterus natalensis*	Museum voucher	North West	–24.76622, 26.37098	Madikwe Game Reserve
UP 0202, TM48026	2007 Dec 01	*Miniopterus natalensis*	Museum voucher	North West	–24.76622, 26.37098	Madikwe Game Reserve

Laboratory no.*	Collection date	Bat species†	ID method	Province	Coordinates	SIte
UP 0203, TM48027	2007 Dec 01	*Scotophilus dinganii*	Museum voucher	North West	–24.76622, 26.32628	Madikwe Game Reserve
UP 0204, TM48028	2007 Dec 01	*Neoromicia (Laephotis) capensis*	Museum voucher	North West	–24.79699, 26.30092	Madikwe Game Reserve
UP 0205, TM48029	2007 Dec 01	*Sauromys petrophilus*	Museum voucher	North West	–24.79699, 26.30092	Madikwe Game Reserve
UP 0206, TM48030	2007 Dec 01	*Neoromicia (Laephotis) capensis*	Museum voucher	North West	–24.79699, 26.30092	Madikwe Game Reserve
UP 0207, TM48031	2007 Dec 01	*Neoromicia (Laephotis) capensis*	Museum voucher	North West	–24.79699, 26.30092	Madikwe Game Reserve
UP 0288	2007 Nov 07	*Miniopterus fraterculus*	Genetic	North West	–26.861075, 27.287641	Thabela thabeng
UP 0291	2007 Nov 07	*Miniopterus fraterculus*	Genetic	North West	–26.861075, 27.287641	Thabela thabeng
UP 0310	2008 Feb 24	*Neoromicia (Laephotis) capensis*	Genetic	Mpumalanga	–25.091742, 30.563794	Paardeplaats
UP 0314	2008 Jun 10	*Chaerephon (Mops) pumilus*	Morphologic	KwaZulu-Natal	-30.203816, 30.780837	Umkomaas
UP 0473	2007 May 10	*Mops condylurus*	Morphologic	Gauteng	–26.397163, 28.463172	Nigel
UP 0639	2010 Sep 03	*Neoromicia (Laephotis) capensis*	Genetic	Gauteng	–25.823356, 28.084432	Hennopsrivier
UP 0713; TM48494	2010 Feb 01	*Glauconycteris (variegate*	Genetic, Museum voucher	Limpopo	–22.42151, 31.2238	Kruger National Park
UP 0716; TM48497	2010 Feb 01	*Scotophilus dinganii*	Museum voucher	Limpopo	–22.42151, 31.2238	Kruger National Park
UP 0717; TM48498	2010 Feb 01	*Nycticeinops schlieffeni*	Museum voucher	Limpopo	–22.42151, 31.2238	Kruger National Park
UP 0719; TM48500	2010 Feb 01	*Nycticeinops schlieffeni*	Museum voucher	Limpopo	–22.42151, 31.2238	Kruger National Park
UP 0720; TM48501	2010 Feb 01	*Nycticeinops schlieffeni*	Museum voucher	Limpopo	–22.42151, 31.2238	Kruger National Park
UP 0721; TM48502	2010 Feb 01	*Nycticeinops schlieffeni*	Museum voucher	Limpopo	–22.42151, 31.2238	Kruger National Park
UP 0722; TM48503	2010 Feb 01	*Scotophilus dinganii*	Museum voucher	Limpopo	–22.42151, 31.2238	Kruger National Park
UP 0723; TM48504	2010 Feb 01	*Scotophilus dinganii*	Museum voucher	Limpopo	–22.42151, 31.2238	Kruger National Park
UP 0724; TM48505	2010 Feb 01	*Neoromicia (Laephotis) nana*	Museum voucher	Limpopo	–22.42151, 31.2238	Kruger National Park
UP 0725; TM48506	2010 Feb 01	*Neoromicia (Laephotis) zuluensis*	Museum voucher	Limpopo	–22.42151, 31.2238	Kruger National Park
UP 0726; TM48507	2010 Feb 01	*Myotis tricolor*	Museum voucher	Limpopo	–22.42151, 31.2238	Kruger National Park
UP 0728; TM48509	2010 Feb 01	*Neoromicia (Laephotis) nana*	Museum voucher	Limpopo	–22.42151, 31.2238	Kruger National Park
UP 0733; TM48512	2010 Feb 02	*Mops condylurus*	Museum voucher	Limpopo	–22.42601, 31.2987	Kruger National Park
UP 0734; TM48513	2010 Feb 02	*Mops condylurus*	Museum voucher	Limpopo	–22.42601, 31.2987	Kruger National Park
UP 0739; TM48518	2010 Feb 02	*Scotophilus dinganii*	Museum voucher	Limpopo	–22.42601, 31.2987	Kruger National Park
UP 0741; TM48502	2010 Feb 02	*Scotophilus dinganii*	Museum voucher	Limpopo	–22.42601, 31.2987	Kruger National Park
UP 0745; TM48523	2010 Feb 02	*Pipistrellus rusticus*	Museum voucher	Limpopo	–22.42601, 31.2987	Kruger National Park
UP 0746; TM48524	2010 Feb 02	*Pipistrellus rusticus*	Museum voucher	Limpopo	–22.42601, 31.2987	Kruger National Park
UP 0747; TM48525	2010 Feb 02	*Pipistrellus rusticus*	Museum voucher	Limpopo	–22.42601, 31.2987	Kruger National Park

Laboratory no.*	Collection date	Bat species†	ID method	Province	Coordinates	SIte
UP 0748; TM48526	2010 Feb 02	*Pipistrellus rusticus*	Museum voucher	Limpopo	–22.42601, 31.2987	Kruger National Park
UP 0749; TM48527	2010 Feb 02	*Pipistrellus rusticus*	Museum voucher	Limpopo	–22.42601, 31.2987	Kruger National Park
UP 0755; TM48528	2010 Feb 03	*Taphozous mauritianus*	Museum voucher	Limpopo	–22.34657, 31.11595	Kruger National Park
UP 0758; TM48531	2010 Feb 03	*Chaerephon (Mops) pumilus*	Museum voucher	Limpopo	–22.34657, 31.11595	Kruger National Park
UP 0759; TM48532	2010 Feb 03	*Chaerephon (Mops) pumilus*	Museum voucher	Limpopo	–22.34657, 31.11595	Kruger National Park
UP 0761; TM48534	2010 Feb 03	*Neoromicia (Laephotis) nana*	Museum voucher	Limpopo	–22.34657, 31.11595	Kruger National Park
UP 0762; TM48535	2010 Feb 03	*Neoromicia (Laephotis)* cf. *helios*	Museum voucher	Limpopo	–22.34657, 31.11595	Kruger National Park
UP 0763; TM48536	2010 Feb 03	*Neoromicia (Laephotis)* cf. *helios*	Museum voucher	Limpopo	–22.34657, 31.11595	Kruger National Park
UP 0764; TM48537	2010 Feb 03	*Neoromicia (Laephotis)* cf. *helios*	Museum voucher	Limpopo	–22.34657, 31.11595	Kruger National Park
UP 0766; TM48539	2010 Feb 03	*Scotophilus dinganii*	Genetic, Museum voucher	Limpopo	–22.34657, 31.11595	Kruger National Park
UP 0767; TM48540	2010 Feb 03	*Scotophilus dinganii*	Museum voucher	Limpopo	–22.34657, 31.11595	Kruger National Park
UP 0768; TM48541	2010 Feb 03	*Mops condylurus*	Museum voucher	Limpopo	–22.34657, 31.11595	Kruger National Park
UP 0769; TM48542	2010 Feb 03	*Taphozous mauritianus*	Museum voucher	Limpopo	–22.34657, 31.11595	Kruger National Park
UP 0770; TM48543	2010 Feb 03	*Mops condylurus*	Museum voucher	Limpopo	–22.34657, 31.11595	Kruger National Park
UP 0771; TM48544	2010 Feb 03	*Mops condylurus*	Museum voucher	Limpopo	–22.34657, 31.11595	Kruger National Park
UP 0772; TM48545	2010 Feb 03	*Chaerephon (Mops) ansorgei*	Museum voucher	Limpopo	–22.34657, 31.11595	Kruger National Park
UP 0774; TM48547	2010 Feb 03	*Nycticeinops schlieffeni*	Museum voucher	Limpopo	–22.34657, 31.11595	Kruger National Park
UP 0775; TM48548	2010 Feb 03	*Nycticeinops schlieffeni*	Museum voucher	Limpopo	–22.34657, 31.11595	Kruger National Park
UP 0779; TM48552	2010 Feb 03	*Scotophilus viridis*	Museum voucher	Limpopo	–22.34657, 31.11595	Kruger National Park
UP 0780; TM48553	2010 Feb 03	*Scotophilus leucogaster*	Genetic, Museum voucher	Limpopo	–22.34657, 31.11595	Kruger National Park
UP 0781; TM48554	2010 Feb 03	*Scotophilus viridis*	Museum voucher	Limpopo	–22.34657, 31.11595	Kruger National Park
UP 0782; TM48555	2010 Feb 03	*Scotophilus viridis*	Museum voucher	Limpopo	–22.34657, 31.11595	Kruger National Park
UP 0783; TM48556	2010 Feb 03	*Scotophilus viridis*	Museum voucher	Limpopo	–22.34657, 31.11595	Kruger National Park
UP 0784; TM48557	2010 Feb 03	*Scotophilus viridis*	Museum voucher	Limpopo	–22.34657, 31.11595	Kruger National Park
UP 0785; TM48558	2010 Feb 03	*Scotophilus leucogaster*	Museum voucher	Limpopo	–22.34657, 31.11595	Kruger National Park
UP 0786; TM48559	2010 Feb 03	*Scotophilus leucogaster*	Museum voucher	Limpopo	–22.34657, 31.11595	Kruger National Park
UP 0787; TM48560	2010 Feb 03	*Scotophilus leucogaster*	Museum voucher	Limpopo	–22.34657, 31.11595	Kruger National Park
UP 0827; TM48611	2010 Apr 12	*Glauconycteris variegata*	Museum voucher	KwaZulu-Natal	–27.262925, 32.768556	Rocktail Beach Camp
UP 0828; TM48612	2010 Apr 14	*Nycteris thebaica*	Museum voucher	KwaZulu-Natal	–27.221520, 32.793200	Rocktail Beach Camp
UP 0829; TM48613	2010 Apr 14	*Nycteris thebaica*	Museum voucher	KwaZulu-Natal	–27.221520, 32.793200	Rocktail Beach Camp
UP 0830; TM48614	2010 Apr 13	*Scotophilus dinganii*	Museum voucher	KwaZulu-Natal	–27.221520, 32.793200	Rocktail Beach Camp

Laboratory no.*	Collection date	Bat species†	ID method	Province	Coordinates	SIte
UP 0831; TM48615	2010 Apr 13	*Scotophilus dinganii*	Museum voucher	KwaZulu-Natal	–27.221520, 32.793200	Rocktail Beach Camp
UP 0833; TM48617	2010 Apr 13	*Scotophilus dinganii*	Museum voucher	KwaZulu-Natal	–27.221520, 32.793200	Rocktail Beach Camp
UP 0834; TM48618	2010 Apr 13	*Scotophilus dinganii*	Museum voucher	KwaZulu-Natal	–27.221520, 32.793200	Rocktail Beach Camp
UP 0835; TM48619	2010 Apr 13	*Chaerephon (Mops) pumilus*	Museum voucher	KwaZulu-Natal	–27.221520, 32.793200	Rocktail Beach Camp
UP 0836; TM48620	2010 Apr 13	*Chaerephon (Mops) pumilus*	Museum voucher	KwaZulu-Natal	–27.221520, 32.793200	Rocktail Beach Camp
UP 0837; TM48621	2010 Apr 13	*Chaerephon (Mops) pumilus*	Museum voucher	KwaZulu-Natal	–27.221520, 32.793200	Rocktail Beach Camp
UP 0838; TM48622	2010 Apr 13	*Chaerephon (Mops) pumilus*	Museum voucher	KwaZulu-Natal	–27.221520, 32.793200	Rocktail Beach Camp
UP 0839; TM48623	2010 Apr 14	*Chaerephon (Mops) pumilus*	Museum voucher	KwaZulu-Natal	–27.221520, 32.793200	Rocktail Beach Camp
UP 0840; TM48624	2010 Apr 13	*Pipistrellus hesperidus*	Museum voucher	KwaZulu-Natal	–27.221520, 32.793200	Rocktail Beach Camp
UP 0842; TM48626	2010 Apr 13	*Pipistrellus hesperidus*	Museum voucher	KwaZulu-Natal	–27.221520, 32.793200	Rocktail Beach Camp
UP 0843; TM48627	2010 Apr 14	*Nycteris thebaica*	Museum voucher	KwaZulu-Natal	–27.221520, 32.793200	Rocktail Beach Camp
UP 0844; TM48628	2010 Apr 14	*Kerivoula (Glauconycteris) argentata*	Museum voucher	KwaZulu-Natal	–27.221520, 32.793200	Rocktail Beach Camp
UP 0845; TM48629	2010 Apr 14	*Scotophilus dinganii*	Museum voucher	KwaZulu-Natal	–27.262925, 32.76855	Rocktail Beach Camp
UP 0846; TM48630	2010 Apr 14	*Glauconycteris variegata*	Museum voucher	KwaZulu-Natal	–27.262925, 32.76855	Rocktail Beach Camp
UP 0847; TM48631	2010 Apr 14	*Scotophilus dinganii*	Museum voucher	KwaZulu-Natal	–27.262925, 32.76855	Rocktail Beach Camp
UP 0848; TM48632	2010 Apr 14	*Scotophilus dinganii*	Museum voucher	KwaZulu-Natal	–27.262925, 32.76855	Rocktail Beach Camp
UP 0854; TM48635	2010 Apr 15	*Nycteris thebaica*	Museum voucher	KwaZulu-Natal	–27.22152, 32.7932	Rocktail Beach Camp
UP 0855; TM48636	2010 Apr 15	*Nycteris thebaica*	Museum voucher	KwaZulu-Natal	–27.22152, 32.7932	Rocktail Beach Camp
UP 0856; TM48637	2010 Apr 15	*Nycteris thebaica*	Museum voucher	KwaZulu-Natal	–27.22152, 32.7932	Rocktail Beach Camp
UP 0865; TM48638	2010 Apr 15	*Scotophilus dinganii*	Museum voucher	KwaZulu-Natal	–27.262925, 32.768550	Rocktail Beach Camp
UP 0866; TM48639	2010 Apr 15	*Scotophilus dinganii*	Museum voucher	KwaZulu-Natal	–27.262925, 32.768550	Rocktail Beach Camp
UP 0867; TM48640	2010 Apr 15	*Scotophilus dinganii*	Museum voucher	KwaZulu-Natal	–27.262925, 32.768550	Rocktail Beach Camp
UP 0868; TM48641	2010 Apr 15	*Glauconycteris variegata*	Museum voucher	KwaZulu-Natal	–27.262925, 32.768550	Rocktail Beach Camp
UP 0869; TM48642	2010 Apr 15	*Pipistrellus hesperidus*	Museum voucher	KwaZulu-Natal	–27.262925, 32.768550	Rocktail Beach Camp
UP 0870; TM48643	2010 Apr 15	*Pipistrellus hesperidus*	Museum voucher	KwaZulu-Natal	–27.262925, 32.768550	Rocktail Beach Camp
UP 0871; TM48644	2010 Apr 15	*Pipistrellus hesperidus*	Museum voucher	KwaZulu-Natal	–27.262925, 32.768550	Rocktail Beach Camp
UP 0904	2009 Jul 18	*Miniopterus fraterculus*	Genetic	Gauteng	–25.897071, 28.221455	Grootboom Cave, Irene
UP 0912; TM48567	2010 Aug 11	*Hipposideros caffer*	Museum voucher	Limpopo	–22.42151, 31.2238	Kruger National Park
UP 0913; TM48568	2010 Aug 11	*Nycticeinops schlieffeni*	Museum voucher	Limpopo	–22.42151, 31.2238	Kruger National Park
UP 0914; TM48569	2010 Aug 11	*Nycticeinops schlieffeni*	Museum voucher	Limpopo	–22.42151, 31.2238	Kruger National Park
UP 0915; TM48570	2010 Aug 11	*Neoromicia (Laephotis) capensis*	Museum voucher	Limpopo	–22.42151, 31.2238	Kruger National Park
UP 0920; TM48571	2010 Sep 11	*Neoromicia (Laephotis) nana*	Museum voucher	Limpopo	–22.42151, 31.2238	Kruger National Park

Laboratory no.*	Collection date	Bat species†	ID method	Province	Coordinates	SIte
UP 0921; voucher in Kruger National Park collection	2010 Sep 11	*Neoromicia (Laephotis)* cf. *helios*	Morphologic	Limpopo	–22.42151, 31.2238	Kruger National Park
UP 0922; TM48572	2010 Sep 11	*Neoromicia (Laephotis) nana*	Museum voucher	Limpopo	–22.42151, 31.2238	Kruger National Park
UP 0923; TM48573	2010 Sep 11	*Neoromicia (Laephotis) nana*	Museum voucher	Limpopo	–22.42151, 31.2238	Kruger National Park
UP 0929; TM48576	2010 Oct 11	*Scotophilus dinganii*	Museum voucher	Limpopo	–22.42601, 31.29873	Kruger National Park
UP 0930; TM48577	2010 Oct 11	*Scotophilus dinganii*	Museum voucher	Limpopo	–22.42601, 31.29873	Kruger National Park
UP 0931; TM48578	2010 Oct 11	*Neoromicia (Laephotis) helios*	Museum voucher	Limpopo	–22.42601, 31.29873	Kruger National Park
UP 0932; TM48579	2010 Oct 11	*Pipistrellus rusticus*	Museum voucher	Limpopo	–22.42601, 31.29873	Kruger National Park
UP 0934; TM48581	2010 Oct 11	*Chaerephon (Mops) pumilus*	Museum voucher	Limpopo	–22.42601, 31.29873	Kruger National Park
UP 0947; TM48582	2010 Nov 11	*Rhinolophus landeri*	Museum voucher	Limpopo	–22.42601, 31.29873	Kruger National Park
UP 0948; TM48583	2010 Nov 11	*Hipposideros caffer*	Museum voucher	Limpopo	–22.42151, 31.2238	Kruger National Park
UP 0949; TM48584	2010 Dec 11	*Hipposideros caffer*	Museum voucher	Limpopo	–22.34657, 31.11595	Kruger National Park
UP 0951; TM48586	2010 Dec 11	*Hipposideros caffer*	Museum voucher	Limpopo	–22.42151, 31.2238	Kruger National Park
UP 0952; TM48587	2010 Dec 11	*Neoromicia (Laephotis) helios*	Museum voucher	Limpopo	–22.34657, 31.11595	Kruger National Park
UP 0953; TM48588	2010 Dec 11	*Taphozous mauritianus*	Museum voucher	Limpopo	–22.34657, 31.11595	Kruger National Park
UP 0954; TM48589	2010 Dec 11	*Scotophilus viridis*	Museum voucher	Limpopo	–22.34657, 31.11595	Kruger National Park
UP 0955; TM48590	2010 Dec 11	*Scotophilus dinganii*	Museum voucher	Limpopo	–22.34657, 31.11595	Kruger National Park
UP 0956; TM48591	2010 Dec 11	*Scotophilus dinganii*	Museum voucher	Limpopo	–22.34657, 31.11595	Kruger National Park
UP 0957; TM48592	2010 Dec 11	*Scotophilus viridis*	Museum voucher	Limpopo	–22.34657, 31.11595	Kruger National Park
UP 0958; TM48593	2010 Dec 11	*Scotophilus dinganii*	Museum voucher	Limpopo	–22.34657, 31.11595	Kruger National Park
UP 0959; TM48594	2010 Dec 11	*Scotophilus dinganii*	Museum voucher	Limpopo	–22.34657, 31.11595	Kruger National Park
UP 0960; TM48595	2010 Dec 11	*Scotophilus leucogaster*	Museum voucher	Limpopo	–22.34657, 31.11595	Kruger National Park
UP 0961; TM48596	2010 Dec 11	*Neoromicia (Laephotis) zuluensis*	Museum voucher	Limpopo	–22.34657, 31.11595	Kruger National Park
UP 0962; TM48597	2010 Dec 11	*Neoromicia (Laephotis) nana*	Museum voucher	Limpopo	–22.34657, 31.11595	Kruger National Park
UP 0963; TM48598	2010 Dec 11	*Neoromicia (Laephotis) capensis*	Museum voucher	Limpopo	–22.34657, 31.11595	Kruger National Park
UP 0964; TM48599	2010 Dec 11	*Neoromicia (Laephotis) rendalli*	Museum voucher	Limpopo	–22.34657, 31.11595	Kruger National Park
UP 0965; TM48600	2010 Dec 11	*Chaerephon (Mops)ansorgei*	Museum voucher	Limpopo	–22.34657, 31.11595	Kruger National Park
UP 0966; TM48601	2010 Dec 11	*Mops condylurus*	Museum voucher	Limpopo	–22.34657, 31.11595	Kruger National Park
UP 0967; TM48602	2010 Dec 11	*Mops condylurus*	Museum voucher	Limpopo	–22.34657, 31.11595	Kruger National Park
UP 0968; TM48603	2010 Dec 11	*Mops condylurus*	Museum voucher	Limpopo	–22.34657, 31.11595	Kruger National Park
UP 1007; TM48653	2010 Dec 10	*Tadarida aegyptiaca*	Museum voucher	North West	–25.73903, 27.23011	Kgaswane Nature Reserve
UP 1008; TM48646	2010 Dec 10	*Scotophilus dinganii*	Museum voucher	North West	–25.73903, 27.23011	Kgaswane Nature Reserve

Laboratory no.*	Collection date	Bat species†	ID method	Province	Coordinates	SIte
UP 1009; TM48651	2010 Dec 10	*Scotophilus dinganii*	Museum voucher	North West	–25.73903, 27.23011	Kgaswane Nature Reserve
UP 1010; TM48650	2010 Dec 10	*Scotophilus dinganii*	Museum voucher	North West	–25.73903, 27.23011	Kgaswane Nature Reserve
UP 1011; TM48658	2010 Dec 10	*Pipistrellus rusticus*	Museum voucher	North West	–25.73903, 27.23011	Kgaswane Nature Reserve
UP 1012; TM48660	2010 Dec 10	*Neoromicia (Laephotis) capensis*	Museum voucher	North West	–25.73903, 27.23011	Kgaswane Nature Reserve
UP 1013; TM48656	2010 Dec 10	*Scotophilus dinganii*	Museum voucher	North West	–25.73903, 27.23011	Kgaswane Nature Reserve
UP 1014; TM48654	2010 Dec 10	*Scotophilus dinganii*	Museum voucher	North West	–25.73903, 27.23011	Kgaswane Nature Reserve
UP 1015; TM48648	2010 Dec 10	*Scotophilus dinganii*	Museum voucher	North West	–25.73903, 27.23011	Kgaswane Nature Reserve
UP 1016; TM48645	2010 Dec 10	*Scotophilus dinganii*	Museum voucher	North West	–25.73903, 27.23011	Kgaswane Nature Reserve
UP 1017; TM48649	2010 Dec 10	*Scotophilus dinganii*	Museum voucher	North West	–25.73903, 27.23011	Kgaswane Nature Reserve
UP 1018; TM48659	2010 Dec 10	*Scotophilus dinganii*	Genetic, Museum voucher	North West	–25.73903, 27.23011	Kgaswane Nature Reserve
UP 1019; TM48652	2010 Dec 10	*Scotophilus dinganii*	Museum voucher	North West	–25.73903, 27.23011	Kgaswane Nature Reserve
UP 1020; TM48657	2010 Dec 10	*Scotophilus dinganii*	Museum voucher	North West	–25.73903, 27.23011	Kgaswane Nature Reserve
UP 1022; TM48647	2010 Dec 10	*Pipistrellus rusticus*	Genetic, Museum voucher	North West	–25.73903, 27.23011	Kgaswane Nature Reserve
UP 1052; TM48664	2010 Dec 11	*Pipistrellus rusticus*	Museum voucher	North West	–25.7202, 27.18533	Kgaswane Nature Reserve
UP 1054	2010 Dec 03	*Neoromicia (Laephotis) capensis*	Genetic	Gauteng	–25.736022, 28.190374	Pretoria Zoo
UP 1055	2010 Dec 01	*Neoromicia (Laephotis) capensis*	Genetic	Gauteng	–25.736022, 28.190374	Pretoria Zoo
UP 1056	2010 Dec 01	*Tadarida aegyptiaca*	Genetic	Gauteng	–25.736022, 28.190374	Pretoria Zoo
UP 1363	2011 Sep 13	*Scotophilus dinganii*	Morphologic	Limpopo	–23.03262, 29.92965	71 Kameel Street, Louis Trichardt
UP 1364	2011 Sep 13	*Mops midas*	Morphologic	Limpopo	–23.03262, 29.92965	71 Kameel Street, Louis Trichardt
UP 1366	2011 Sep 14	*Rhinolophus simulator*	Morphologic	Limpopo	Not recorded	Labuschagne Farm, Louis Trichardt
UP 1367	2011 Sep 14	*Rhinolophus clivosus*	Morphologic	Limpopo	Not recorded	Labuschagne Farm, Louis Trichardt
UP 1368	2011 Sep 14	*Rhinolophus hildebrandtii s.l.*	Morphologic	Limpopo	Not recorded	Labuschagne Farm, Louis Trichardt
UP 1369	2011 Sep 24	*Neoromicia (Laephotis) capensis*	Genetic	Gauteng	–26.0147. 28.0222	FreeMe Wildlife Rehabilitation Centre
UP 1370	2010 Mar 13	*Miniopterus natalensis*	Morphologic	Gauteng	–25.897071, 28.221455	Grootboom Cave, Irene
UP 1371	2010 Mar 13	*Miniopterus natalensis*	Morphologic	Gauteng	–25.897071, 28.221455	Grootboom Cave, Irene
UP 1372	2010 Mar 13	*Miniopterus natalensis*	Morphologic	Gauteng	–25.897071, 28.221455	Grootboom Cave, Irene
UP 1373	2010 Oct 01	*Miniopterus natalensis*	Genetic	Gauteng	–25.897071, 28.221455	Grootboom Cave, Irene
UP 1374	2011 Apr 01	*Miniopterus natalensis*	Morphologic	Gauteng	–25.897071, 28.221455	Grootboom Cave, Irene
UP 1376	2011 Apr 01	*Miniopterus natalensis*	Morphologic	Gauteng	–25.897071, 28.221455	Grootboom Cave, Irene
UP 1378	2011 Apr 01	*Miniopterus natalensis*	Morphologic	Gauteng	–25.897071, 28.221455	Grootboom Cave, Irene
UP 1382	2011 May 06	*Miniopterus natalensis*	Morphologic	Gauteng	–25.897071, 28.221455	Grootboom Cave, Irene

Laboratory no.*	Collection date	Bat species†	ID method	Province	Coordinates	SIte
UP 1383	2011 May 06	*Miniopterus natalensis*	Morphologic	Gauteng	–25.897071, 28.221455	Grootboom Cave, Irene
UP 1384	2011 May 06	*Miniopterus natalensis*	Morphologic	Gauteng	–25.897071, 28.221455	Grootboom Cave, Irene
UP 1385	2011 May 06	*Miniopterus natalensis*	Morphologic	Gauteng	–25.897071, 28.221455	Grootboom Cave, Irene
UP 1386	2011 May 06	*Miniopterus natalensis*	Morphologic	Gauteng	–25.897071, 28.221455	Grootboom Cave, Irene
UP 1387	2011 May 06	*Miniopterus natalensis*	Morphologic	Gauteng	–25.897071, 28.221455	Grootboom Cave, Irene
UP 1389	2011 Jun 03	*Miniopterus natalensis*	Morphologic	Gauteng	–25.897071, 28.221455	Grootboom Cave, Irene
UP 1390	2011 Jun 03	*Miniopterus natalensis*	Morphologic	Gauteng	–25.897071, 28.221455	Grootboom Cave, Irene
UP 1391	2011 Jun 03	*Miniopterus natalensis*	Morphologic	Gauteng	–25.897071, 28.221455	Grootboom Cave, Irene
UP 1392	2011 Jun 03	*Miniopterus natalensis*	Morphologic	Gauteng	–25.897071, 28.221455	Grootboom Cave, Irene
UP 1395	2011 Jun 29	*Miniopterus natalensis*	Morphologic	Gauteng	–25.897071, 28.221455	Grootboom Cave, Irene
UP 1396	2011 Jun 29	*Miniopterus natalensis*	Morphologic	Gauteng	–25.897071, 28.221455	Grootboom Cave, Irene
UP 1397	2011 Jun 29	*Miniopterus natalensis*	Morphologic	Gauteng	–25.897071, 28.221455	Grootboom Cave, Irene
UP 1398	2011 Jun 29	*Miniopterus natalensis*	Morphologic	Gauteng	–25.897071, 28.221455	Grootboom Cave, Irene
UP 1399	2011 Jun 29	*Miniopterus natalensis*	Morphologic	Gauteng	–25.897071, 28.221455	Grootboom Cave, Irene
UP 1421	2011 Nov 27	*Neoromicia (Laephotis) capensis*	Genetic	Gauteng	Not recorded	Not recorded
UP 1422	2011 Dec 05	*Neoromicia (Laephotis) capensis*	Genetic	Gauteng	Not recorded	Not recorded
UP 1423	2011 Dec 17	*Pipistrellus rusticus*	Morphologic	Gauteng	Not recorded	Not recorded
UP 1424	2011 Jul 25	*Neoromicia (Laephotis) capensis*	Genetic	Gauteng	Not recorded	Not recorded
UP 1425	2011 Jul 25	*Neoromicia (Laephotis) capensis*	Genetic	Gauteng	Not recorded	Not recorded
UP 1426	2011 Jul 25	*Scotophilus dinganii*	Genetic	Gauteng	Not recorded	Not recorded
UP 1427	2011 Jul 25	*Scotophilus dinganii*	Genetic	Gauteng	Not recorded	Not recorded
UP 1428	2012 Apr 21	*Neoromicia (Laephotis) capensis*	Genetic	Gauteng	–25.736022, 28.190374	Pretoria Zoo
UP 1485	2012 Jun 12	*Hipposideros caffer*	Morphologic	Limpopo	–24.11487, 30.12151	Matlapitsi Cave
UP 1486	2012 Jun 12	*Hipposideros caffer*	Morphologic	Limpopo	–24.11487, 30.12151	Matlapitsi Cave
UP 1487	2012 Jun 12	*Miniopterus natalensis*	Morphologic	Limpopo	–24.11487, 30.12151	Matlapitsi Cave
UP 1488	2012 Jun 12	*Miniopterus natalensis*	Morphologic	Limpopo	–24.11487, 30.12151	Matlapitsi Cave
UP 1489	2012 Jun 12	*Hipposideros caffer*	Morphologic	Limpopo	–24.11487, 30.12151	Matlapitsi Cave
UP 1490	2012 Jun 12	*Miniopterus natalensis*	Morphologic	Limpopo	–24.11487, 30.12151	Matlapitsi Cave
UP 1491	2012 Jun 12	*Miniopterus natalensis*	Morphologic	Limpopo	–24.11487, 30.12151	Matlapitsi Cave
UP 1492	2012 Jun 12	*Miniopterus natalensis*	Morphologic	Limpopo	–24.11487, 30.12151	Matlapitsi Cave
UP 1493	2012 Jun 12	*Hipposideros caffer*	Morphologic	Limpopo	–24.11487, 30.12151	Matlapitsi Cave

Laboratory no.*	Collection date	Bat species†	ID method	Province	Coordinates	SIte
UP 1494	2012 Jun 12	*Hipposideros caffer*	Morphologic	Limpopo	–24.11487, 30.12151	Matlapitsi Cave
UP 1495	2012 Jun 12	*Hipposideros caffer*	Morphologic	Limpopo	–24.11487, 30.12151	Matlapitsi Cave
UP 1540	2012 Aug 19	*Nycteris thebaica*	Morphologic	Limpopo	–24.777490, 27.734082	Rooiberg
UP 1547	2012 Sep 20	*Tadarida aegyptiaca*	Genetic	Gauteng	–25.736022, 28.190374	Pretoria Zoo
UP 1601	2012 Nov 08	*Miniopterus natalensis*	Morphologic	Limpopo	–24.11487, 30.12151	Matlapitsi Cave
UP 1604	2012 Nov 08	*Miniopterus natalensis*	Morphologic	Limpopo	–24.11487, 30.12151	Matlapitsi Cave
UP 1700; NHCPHE MAM–29	2013 Jan 16	*Rhinolophus simulator*	Museum voucher	Limpopo	–24.58943, 27.69429	Donkerpoort 448
UP 1702; NHCPHE MAM-45	2013 Jan 16	*Miniopterus natalensis*	Museum voucher	Limpopo	–24.58943, 27.69429	Donkerpoort 448
UP 1703; TM50559	2013 Jan 17	*Miniopterus natalensis*	Museum voucher	Limpopo	–24.58943, 27.69429	Donkerpoort 448
UP 1717; NHCPHE MAM-30	2013 Jan 18	*Miniopterus natalensis*	Museum voucher	Limpopo	–24.58943, 27.69429	Donkerpoort 448
UP 1718; TM50560	2013 Jan 18	*Miniopterus natalensis*	Museum voucher	Limpopo	–24.58941, 27.68473	Randstephne 455
UP 1719; NHCPHE MAM-31	2013 Jan 18	*Miniopterus natalensis*	Museum voucher	Limpopo	–24.58941, 27.68473	Randstephne 455
UP 1723; TM50561	2013 Jan 18	*Miniopterus natalensis*	Museum voucher	Limpopo	–24.58941, 27.68473	Randstephne 455
UP 1731; NHCPHE MAM-46	2013 Jan 18	*Miniopterus natalensis*	Museum voucher	Limpopo	–24.58941, 27.68473	Randstephne 455
UP 1764; NHCPHE MAM-47	2013 Jan 20	*Miniopterus natalensis*	Museum voucher	Limpopo	–24.59751, 27.67145	Randstephne 455
UP 1786; NHCPHE MAM-32	2013 Jan 20	*Miniopterus natalensis*	Museum voucher	Limpopo	–24.59751, 27.67145	Randstephne 455
UP 1787; TM49186	2013 Jan 19	*Neoromicia (Laeophotis) capensis*	Genetic, Museum voucher	Limpopo	–24.59751, 27.67145	Randstephne 455
UP 1788; TM49187	2013 Jan 19	*Pipistrellus rusticus*	Museum voucher	Limpopo	–24.59751, 27.67145	Randstephne 455
UP 1790; TM50558	2013 Jan 20	*Rhinolophus simulator*	Museum voucher	Limpopo	–24.59751, 27.67145	Randstephne 455
UP 1791; TM50589	2013 Jan 20	*Rhinolophus simulator*	Museum voucher	Limpopo	–24.59751, 27.67145	Randstephne 455
UP 1792; TM50554	2013 Jan 20	*Scotophilus dinganii*	Museum voucher	Limpopo	–24.59751, 27.67145	Randstephne 455
UP 1793; TM50555	2013 Jan 20	*Scotophilus dinganii*	Museum voucher	Limpopo	–24.59751, 27.67145	Randstephne 455
UP 1794; NHCPHE MAM–28	2013 Jan 20	*Scotophilus dinganii*	Museum voucher	Limpopo	–24.59751, 27.67145	Randstephne 455
UP 1795; TM50553	2013 Jan 20	*Scotophilus dinganii*	Museum voucher	Limpopo	–24.59751, 27.67145	Randstephne 455
UP 1808; TM50551	2013 Jan 22	*Nycteris thebaica*	Museum voucher	Limpopo	–24.6181, 27.65231	Madimatle Cave
UP 1810; TM50552	2013 Jan 22	*Nycteris thebaica*	Museum voucher	Limpopo	–24.6181, 27.65231	Madimatle Cave
UP 1811; NHCPHE MAM–27	2013 Jan 22	*Nycteris thebaica*	Genetic, Museum voucher	Limpopo	–24.6181, 27.65231	Madimatle Cave
UP 1816, TM48672	2013 Feb 05	*Cloeotis percivali*	Museum voucher	Limpopo	–24.11487, 30.12151	Matlapitsi Cave
UP 1862, TM48673	2013 Feb 05	*Rhinolophus simulator*	Museum voucher	Limpopo	–24.11487, 30.12151	Matlapitsi Cave

Laboratory no.*	Collection date	Bat species†	ID method	Province	Coordinates	SIte
UP 1863, TM48674	2013 Feb 05	*Cloeotis percivali*	Museum voucher	Limpopo	–24.11487, 30.12151	Matlapitsi Cave
UP 1864, TM48675	2013 Feb 05	*Rhinolophus darlingi*	Museum voucher	Limpopo	–24.11487, 30.12151	Matlapitsi Cave
UP 1865, TM48676	2013 Feb 05	*Myotis tricolor*	Museum voucher	Limpopo	–24.11487, 30.12151	Matlapitsi Cave
UP 2002, TM48677	2013 Mar 05	*Hipposideros caffer*	Museum voucher	Limpopo	–24.11487, 30.12151	Matlapitsi Cave
UP 2003, TM48678	2013 Mar 05	*Hipposideros caffer*	Museum voucher	Limpopo	–24.11487, 30.12151	Matlapitsi Cave
UP 2004, TM48679	2013 Mar 05	*Rhinolophus clivosus*	Museum voucher	Limpopo	–24.11487, 30.12151	Matlapitsi Cave
UP 2005, TM48680	2013 Mar 05	*Rhinolophus darlingi*	Museum voucher	Limpopo	–24.11487, 30.12151	Matlapitsi Cave
UP 2006, TM48681	2013 Mar 05	*Rhinolophus clivosus*	Museum voucher	Limpopo	–24.11487, 30.12151	Matlapitsi Cave
UP 2007, TM48682	2013 Mar 05	*Rhinolophus clivosus*	Museum voucher	Limpopo	–24.11487, 30.12151	Matlapitsi Cave
UP 2008, TM48683	2013 Mar 05	*Rhinolophus darlingi*	Museum voucher	Limpopo	–24.11487, 30.12151	Matlapitsi Cave
UP 2009, TM48684	2013 Mar 05	*Rhinolophus darlingi*	Museum voucher	Limpopo	–24.11487, 30.12151	Matlapitsi Cave
UP 2010, TM48685	2013 Mar 05	*Hipposideros caffer*	Museum voucher	Limpopo	–24.11487, 30.12151	Matlapitsi Cave
UP 2011, TM48686	2013 Mar 05	*Hipposideros caffer*	Museum voucher	Limpopo	–24.11487, 30.12151	Matlapitsi Cave
UP 2766, TM50659	2013 Jul 02	*Miniopterus natalensis*	Museum voucher	Limpopo	–24.11487, 30.12151	Matlapitsi Cave
UP 3012, TM50645	2013 Jul 08	*Miniopterus natalensis*	Genetic, Museum voucher	Limpopo	–24.11487, 30.12151	Matlapitsi Cave
UP 3270	2013 Oct 02	*Miniopterus natalensis*	Morphologic	Limpopo	–24.11487, 30.12151	Matlapitsi Cave
UP 3271	2013 Oct 02	*Miniopterus natalensis*	Morphologic	Limpopo	–24.11487, 30.12151	Matlapitsi Cave
UP 3272	2013 Oct 02	*Miniopterus natalensis*	Morphologic	Limpopo	–24.11487, 30.12151	Matlapitsi Cave
UP 3273	2013 Oct 02	*Miniopterus natalensis*	Morphologic	Limpopo	–24.11487, 30.12151	Matlapitsi Cave
UP 3274	2013 Oct 02	*Miniopterus natalensis*	Morphologic	Limpopo	–24.11487, 30.12151	Matlapitsi Cave
UP 3435, TM50662	2013 Nov 05	*Miniopterus natalensis*	Museum voucher	Limpopo	–24.11487, 30.12151	Matlapitsi Cave
UP 3465, TM50658	2013 Nov 05	*Cloeotis percivali*	Museum voucher	Limpopo	–24.11487, 30.12151	Matlapitsi Cave
UP 3563	2013 Nov 26	*Miniopterus natalensis*	Morphologic	Limpopo	–24.11487, 30.12151	Matlapitsi Cave
UP 3566	2013 Nov 26	*Miniopterus natalensis*	Morphologic	Limpopo	–24.11487, 30.12151	Matlapitsi Cave
UP 3567	2013 Nov 26	*Miniopterus natalensis*	Morphologic	Limpopo	–24.11487, 30.12151	Matlapitsi Cave
UP 3568	2013 Nov 26	*Miniopterus natalensis*	Morphologic	Limpopo	–24.11487, 30.12151	Matlapitsi Cave
UP 3569	2013 Nov 26	*Miniopterus natalensis*	Morphologic	Limpopo	–24.11487, 30.12151	Matlapitsi Cave
UP 3610	2013 Nov 26	*Miniopterus natalensis*	Morphologic	Limpopo	–24.11487, 30.12151	Matlapitsi Cave
UP 3664	2013 Nov 27	*Miniopterus natalensis*	Morphologic	Limpopo	–24.11487, 30.12151	Matlapitsi Cave
UP 3665	2013 Nov 27	*Hipposideros caffer*	Morphologic	Limpopo	–24.11487, 30.12151	Matlapitsi Cave
UP 3666	Not recorded	*Tadarida aegyptiaca*	Genetic	Gauteng	–25.751234, 28.276352	Council for Scientific and Industrial Research
UP 3801; TM50567	2014 Jan 20	*Miniopterus natalensis*	Museum voucher	Limpopo	–24.6181, 27.65231	Madimatle Cave
UP 3802; TM50564	2014 Jan 20	*Rhinolophus simulator*	Museum voucher	Limpopo	–24.6181, 27.65231	Madimatle Cave

Laboratory no.*	Collection date	Bat species†	ID method	Province	Coordinates	SIte
UP 3803	2014 Jan 21	*Miniopterus natalensis*	Morphologic	Limpopo	–24.6181, 27.65231	Madimatle Cave
UP 3804; NHCPHE MAM-36	2014 Jan 21	*Miniopterus natalensis*	Museum voucher	Limpopo	–24.6181, 27.65231	Madimatle Cave
UP 3805; TM50568	2014 Jan 21	*Miniopterus natalensis*	Museum voucher	Limpopo	–24.6181, 27.65231	Madimatle Cave
UP 3815; NHCPHE MAM-37	2014 Jan 21	*Nycteris thebaica*	Genetic, Museum voucher	Limpopo	–24.6181, 27.65231	Madimatle Cave
UP 3825; TM50569	2014 Jan 21	*Rhinolophus smithersi*	Museum voucher	Limpopo	–24.6181, 27.65231	Madimatle Cave
UP 3826; NHCPHE MAM-38	2014 Jan 21	*Rhinolophus smithersi*	Museum voucher	Limpopo	–24.6181, 27.65231	Madimatle Cave
UP 3830; TM50570	2014 Jan 21	*Rhinolophus simulator*	Museum voucher	Limpopo	–24.6181, 27.65231	Madimatle Cave
UP 3887, TM49146	2014 Feb 05	*Neoromicia (Laephotis) nana*	Genetic, Museum voucher	Limpopo	–24.11487, 30.12151	Matlapitsi Cave
UP 3917, TM49148	2014 Feb 05	*Pipistrellus hesperidus*	Museum voucher	Limpopo	–24.11487, 30.12151	Matlapitsi Cave
UP 3918, TM48149	2014 Feb 05	*Pipistrellus hesperidus*	Museum voucher	Limpopo	–24.11487, 30.12151	Matlapitsi Cave
UP 3920, TM49168	2014 Feb 11	*Pipistrellus hesperidus*	Morphologic	Mpumalanga	–25.71475, 28.98213	Blaauwpoort 257
UP 3921, TM49169	2014 Feb 12	*Neoromicia (Laephotis) capensis*	Museum voucher	Mpumalanga	–25.7113, 29.01755	Blaauwpoort 257
UP 3922, TM49170	2014 Feb 12	*Neoromicia (Laephotis) capensis*	Museum voucher	Mpumalanga	–25.72596, 28.99048	Blaauwpoort 257
UP 3923, TM49171	2014 Feb 12	*Neoromicia (Laephotis) capensis*	Museum voucher	Mpumalanga	–25.72596, 28.99048	Blaauwpoort 257
UP 3924, TM49172	2014 Feb 12	*Pipistrellus rusticus*	Morphologic	Mpumalanga	–25.72596, 28.99048	Blaauwpoort 257
UP 3925, TM49173	2014 Feb 15	*Neoromicia (Laephotis) zuluensis*	Morphologic	Mpumalanga	–25.65755, 29.01664	Blaauwpoort 257
UP 3926, TM49174	2014 Feb 21	*Rhinolophus clivosus*	Morphologic	Mpumalanga	–25.6862, 29.0121	Blaauwpoort 257
UP 3927, TM49175	2014 Feb 22	*Myotis tricolor*	Morphologic	Mpumalanga	–25.71454, 28.98284	Blaauwpoort 257
UP 3928	2009 Sep 30	*Neoromicia (Laephotis) capensis*	Genetic	Gauteng	–25.736022, 28.190374	Pretoria Zoo
UP 4105, NHCPHE MAM-73	2014 Apr 08	*Rhinolophus clivosus*	Museum voucher	Limpopo	–24.6181, 27.65231	Madimatle Cave
UP 4142, TM50565	2014 May 07	*Rhinolophus simulator*	Museum voucher	Limpopo	–24.6181, 27.65231	Madimatle Cave
UP 4143, NHCPHE MAM-35	2014 May 07	*Rhinolophus simulator*	Museum voucher	Limpopo	–24.6181, 27.65231	Madimatle Cave
UP 4150, TM50566	2014 May 07	*Rhinolophus simulator*	Museum voucher	Limpopo	–24.6181, 27.65231	Madimatle Cave
UP 4165	2014 May 13	*Miniopterus natalensis*	Morphologic	Limpopo	–24.11487, 30.12151	Matlapitsi Cave
UP 4166	2014 May 13	*Miniopterus natalensis*	Genetic	Limpopo	–24.11487, 30.12151	Matlapitsi Cave
UP 4532; TM50590	2014 Sep 06	*Nycteris thebaica*	Museum voucher	Limpopo	–24.6181, 27.65231	Madimatle Cave
UP 4701, TM50636	2014 Nov 28	*Miniopterus natalensis*	Genetic, Museum voucher	Limpopo	–24.59751, 27.67145	Randstephne 455
UP 4702, TM50637	2014 Nov 28	*Miniopterus natalensis*	Museum voucher	Limpopo	–24.59751, 27.67145	Randstephne 455

Laboratory no.*	Collection date	Bat species†	ID method	Province	Coordinates	SIte
UP 4749	2014 Dec 02	*Miniopterus natalensis*	Morphologic	Limpopo	–24.11487, 30.12151	Matlapitsi Cave
UP 4750	2014 Dec 02	*Rhinolophus simulator*	Morphologic	Limpopo	–24.11487, 30.12151	Matlapitsi Cave
UP 4830, TM49113	2015 Dec 01	*Rhinolophus simulator*	Museum voucher	Limpopo	–24.11487, 30.12151	Matlapitsi Cave
UP 4831, TM49150	2015 Feb 01	*Rhinolophus simulator*	Museum voucher	Limpopo	–24.11487, 30.12151	Matlapitsi Cave
UP 4840, TM49151	2015 Feb 01	*Hipposideros caffer*	Museum voucher	Limpopo	–24.11487, 30.12151	Matlapitsi Cave
UP 4845, TM49152	2015 Feb 01	*Myotis tricolor*	Museum voucher	Limpopo	–24.11487, 30.12151	Matlapitsi Cave
UP 4903, TM49160	2015 Feb 02	*Rhinolophus clivosus*	Museum voucher	Limpopo	–24.11487, 30.12151	Matlapitsi Cave
UP 4904, TM49161	2015 Feb 02	*Myotis tricolor*	Museum voucher	Limpopo	–24.11487, 30.12151	Matlapitsi Cave
UP 4908, NHCPHE MAM-50	2015 Feb 23	*Rhinolophus simulator*	Museum voucher	Limpopo	–24.6181, 27.65231	Madimatle Cave
UP 4909, TM50591	2015 Feb 23	*Nycteris thebaica*	Museum voucher	Limpopo	–24.6181, 27.65231	Madimatle Cave
UP 4918, NHCPHE MAM-51	2015 Feb 23	*Rhinolophus blasii*	Museum voucher	Limpopo	–24.6181, 27.65231	Madimatle Cave
UP 4970, NHCPHE MAM-72	2015 Feb 27	*Myotis tricolor*	Museum voucher	Limpopo	–24.6181, 27.65231	Madimatle Cave
UP 4971, NHCPHE MAM-52	2015 Feb 27	*Pipistrellus rusticus*	Museum voucher	Limpopo	–24.6181, 27.65231	Madimatle Cave
UP 4972, TM49189	2015 Feb 28	*Neoromicia (Laephotis) capensis*	Museum voucher	Limpopo	–24.61737, 27.68773	Jonker Trust Farm
UP 4974, TM50592	2015 Feb 27	*Rhinolophus simulator*	Museum voucher	Limpopo	–24.6181, 27.65231	Madimatle Cave
UP 4985, NHCPHE MAM-54	2015 Mar 01	*Rhinolophus blasii*	Museum voucher	Limpopo	–24.6181, 27.65231	Madimatle Cave
UP 4988, TM50593	2015 Mar 01	*Rhinolophus blasii*	Museum voucher	Limpopo	–24.6181, 27.65231	Madimatle Cave
UP 4997, NHCPHE MAM-53	2015 Mar 02	*Rhinolophus simulator*	Museum voucher	Limpopo	–24.6181, 27.65231	Madimatle Cave
UP 5013; TM49069	2015 Mar 04	*Neoromicia (Laephotis) capensis*	Museum voucher	Limpopo	–24.625197, 27.697872	Jonker Trust Farm
UP 5014, TM49190	2015 Mar 04	*Neoromicia (Laephotis) capensis*	Museum voucher	Limpopo	–24.625197, 27.697872	Jonker Trust Farm
UP 5023, TM49164	2015 Apr 21	*Rhinolophus clivosus*	Genetic, Museum voucher	Gauteng	–25.897071, 28.221455	Grootboom Cave, Irene
UP 5024, TM49165	2015 Apr 21	*Rhinolophus clivosus*	Genetic, Museum voucher	Gauteng	–25.897071, 28.221455	Grootboom Cave, Irene
UP 5025, TM49166	2015 Apr 21	*Miniopterus natalensis*	Museum voucher	Gauteng	–25.897071, 28.221455	Grootboom Cave, Irene
UP 5026, TM49167	2015 Apr 21	*Miniopterus natalensis*	Museum voucher	Gauteng	–25.897071, 28.221455	Grootboom Cave, Irene
UP 5027, TM49176	2015 Apr 16	*Rhinolophus clivosus*	Museum voucher	KwaZulu-Natal	–29.48694, 29.89806	Wakefield Farm
UP 5028, TM49177	2015 Apr 17	*Rhinolophus clivosus*	Museum voucher	KwaZulu-Natal	–29.48694, 29.89806	Wakefield Farm
UP 5029, TM49178	2015 Apr 17	*Rhinolophus swinnyi*	Museum voucher	KwaZulu-Natal	–29.48694, 29.89806	Wakefield Farm
UP 5030, TM49179	2015 Apr 17	*Rhinolophus swinnyi*	Museum voucher	KwaZulu-Natal	–29.48694, 29.89806	Wakefield Farm

Laboratory no.*	Collection date	Bat species†	ID method	Province	Coordinates	SIte
UP 5031, TM49180	2015 Apr 17	*Rhinolophus swinnyi*	Museum voucher	KwaZulu-Natal	–29.48694, 29.89806	Wakefield Farm
UP 5035, TM49181	2015 Apr 20	*Laephotis botswanae*	Museum voucher	KwaZulu-Natal	–29.48694, 29.89806	Wakefield Farm
UP 5036, TM49182	2015 Apr 20	*Neoromicia (Laephotis) capensis*	Museum voucher	KwaZulu-Natal	–29.48694, 29.89806	Wakefield Farm
UP 5038, TM49183	2015 Apr 20	*Neoromicia (Laephotis) capensis*	Genetic, Museum voucher	KwaZulu-Natal	–29.48694, 29.89806	Wakefield Farm
UP 5039, TM49184	2015 Apr 21	*Rhinolophus clivosus*	Museum voucher	KwaZulu-Natal	–29.48694, 29.89806	Wakefield Farm
UP 5040, TM49195	2015 Apr 29	*Tadarida aegyptiaca*	Museum voucher	KwaZulu-Natal	–29.48694, 29.89806	Wakefield Farm
UP 5060	2015 Mar 03	*Rhinolophus simulator*	Morphologic	Limpopo	–24.11487, 30.12151	Matlapitsi Cave
UP 5061	2015 Mar 03	*Rhinolophus simulator*	Morphologic	Limpopo	–24.11487, 30.12151	Matlapitsi Cave
UP 5066	2015 Mar 03	*Rhinolophus simulator*	Morphologic	Limpopo	–24.11487, 30.12151	Matlapitsi Cave
UP 5121	2015 Mar 04	*Rhinolophus clivosus*	Morphologic	Limpopo	–24.11487, 30.12151	Matlapitsi Cave
UP 5134	2015 Mar 04	*Miniopterus natalensis*	Genetic	Limpopo	–24.11487, 30.12151	Matlapitsi Cave
UP 5233	2015 May 06	*Miniopterus natalensis*	Genetic	Limpopo	–24.11487, 30.12151	Matlapitsi Cave
UP 5275, TM49163	2015 May 06	*Rhinolophus clivosus*	Museum voucher	Limpopo	–24.11487, 30.12151	Matlapitsi Cave
UP 5279, NHCPHE MAM-74	2015 May 11	*Nycteris thebaica*	Museum voucher	Limpopo	–24.6181, 27.65231	Madimatle Cave
UP 5287, NHCPHE MAM-75	2015 May 11	*Rhinolophus blasii*	Museum voucher	Limpopo	–24.6181, 27.65231	Madimatle Cave
UP 5296, NHCPHE MAM-76	2015 May 11	*Rhinolophus simulator*	Museum voucher	Limpopo	–24.6181, 27.65231	Madimatle Cave
UP 5304; TM49194	2015 May 11	*Neoromicia (Laephotis) capensis*	Museum voucher	KwaZulu-Natal	–29.331947, 30.27753	Welgevonden Farm
UP 5319, NHCPHE MAM-77	2015 May 12	*Nycteris thebaica*	Museum voucher	Limpopo	–24.6181, 27.65231	Madimatle Cave
UP 5431, TM50664	2015 Jul 08	*Miniopterus natalensis*	Museum voucher	Limpopo	–24.11487, 30.12151	Matlapitsi Cave
UP 5449, TM50663	2015 Jul 15	*Miniopterus natalensis*	Museum voucher	Gauteng	–25.897071, 28.221455	Grootboom Cave, Irene
UP 5450, TM50652	2015 Jul 15	*Miniopterus natalensis*	Museum voucher	Gauteng	–25.897071, 28.221455	Grootboom Cave, Irene
UP 5463, TM50594	2015 Jul 20	*Nycteris thebaica*	Museum voucher	Limpopo	–24.6181, 27.65231	Madimatle Cave
UP 5467, NHCPHE MAM-55	2015 Jul 20	*Nycteris thebaica*	Museum voucher	Limpopo	–24.6181, 27.65231	Madimatle Cave
UP 5478, TM50595	2015 Jul 20	*Rhinolophus simulator*	Museum voucher	Limpopo	–24.6181, 27.65231	Madimatle Cave
UP 5491, NHCPHE MAM-16	2015 Jul 21	*Nycteris thebaica*	Genetic, Museum voucher	Limpopo	–24.6181, 27.65231	Madimatle Cave
UP 5608	2015 Sep 02	*Miniopterus natalensis*	Genetic	Limpopo	–24.11487, 30.12151	Matlapitsi Cave
UP 5619	2015 Sep 02	*Miniopterus natalensis*	Genetic	Limpopo	–24.11487, 30.12151	Matlapitsi Cave
UP 5744, NHCPHE MAM-78	2015 Sep 16	*Rhinolophus simulator*	Museum voucher	Limpopo	–24.6181, 27.65231	Madimatle Cave
UP 5784	2015 Sep 21	*Hipposideros caffer*	Morphologic	Mpumalanga	–24.996531, 31.591762	Kruger National Park-

Laboratory no.*	Collection date	Bat species†	ID method	Province	Coordinates	SIte
UP 5830	2015 Oct 27	*Rhinolophus blasii*	Morphologic	Limpopo	–24.11487, 30.12151	Matlapitsi Cave
UP 5842	2015 Oct 27	*Miniopterus natalensis*	Morphologic	Limpopo	–24.11487, 30.12151	Matlapitsi Cave
UP 5846	2015 Oct 27	*Miniopterus natalensis*	Morphologic	Limpopo	–24.11487, 30.12151	Matlapitsi Cave
UP 5927	2015 Oct 26	*Miniopterus natalensis*	Genetic	Limpopo	–24.11487, 30.12151	Matlapitsi Cave
UP 5928	2015 Oct 26	*Miniopterus natalensis*	Morphologic	Limpopo	–24.11487, 30.12151	Matlapitsi Cave
UP 5929	2015 Oct 26	*Miniopterus fraterculus*	Genetic	Limpopo	–24.11487, 30.12151	Matlapitsi Cave
UP 5930	2015 Oct 26	*Miniopterus natalensis*	Morphologic	Limpopo	–24.11487, 30.12151	Matlapitsi Cave
UP 5934	2015 Oct 27	*Miniopterus natalensis*	Morphologic	Limpopo	–24.11487, 30.12151	Matlapitsi Cave
UP 6004, NHCPHE MAM-57	2015 Nov 10	*Nycteris thebaica*	Museum voucher	Limpopo	–24.6181, 27.65231	Madimatle Cave
UP 6076, TM50597	2015 Nov 12	*Cloeotis percivali*	Museum voucher	Limpopo	–24.6181, 27.65231	Madimatle Cave
UP 6080, NHCPHE MAM-58	2015 Nov 12	*Cloeotis percivali*	Museum voucher	Limpopo	–24.6181, 27.65231	Madimatle Cave
UP 6120	2016 Jan 12	*Myotis tricolor*	Morphologic	Limpopo	–24.11487, 30.12151	Matlapitsi Cave
UP 6246, NHCPHE MAM-59	2016 Jan 19	*Miniopterus natalensis*	Genetic, Museum voucher	Limpopo	–24.6181, 27.65231	Madimatle Cave
UP 6247, TM50598	2016 Jan 19	*Miniopterus natalensis*	Museum voucher	Limpopo	–24.6181, 27.65231	Madimatle Cave
UP 6290, TM49236	2016 Jan 20	*Miniopterus natalensis*	Museum voucher	Limpopo	–24.6181, 27.65231	Madimatle Cave
UP 6300, NHCPHE MAM-61	2016 Jan 20	*Nycteris thebaica*	Museum voucher	Limpopo	–24.6181, 27.65231	Madimatle Cave
UP 6302, TM50599	2016 Jan 20	*Miniopterus natalensis*	Museum voucher	Limpopo	–24.6181, 27.65231	Madimatle Cave
UP 6381, TM50572	2016 Apr 05	*Rhinolophus simulator*	Museum voucher	Limpopo	–24.6181, 27.65231	Madimatle Cave
UP 6395, NHCPHE MAM-39	2016 Apr 07	*Rhinolophus simulator*	Museum voucher	Limpopo	–24.6181, 27.65231	Madimatle Cave
UP 6399, TM50571	2016 Apr 07	*Miniopterus natalensis*	Museum voucher	Limpopo	–24.6181, 27.65231	Madimatle Cave
UP 6795, TM50665	2016 Jun 06	*Rhinolophus simulator*	Museum voucher	Limpopo	–24.6181, 27.65231	Madimatle Cave
UP 6814; NHCPHE MAM-17	2016 Jun 07	*Rhinolophus smithersi*	Museum voucher	Limpopo	–24.6181, 27.65231	Madimatle Cave
UP 6826, NHCPHE MAM-42	2016 Jun 08	*Rhinolophus simulator*	Museum voucher	Limpopo	–24.6181, 27.65231	Madimatle Cave
UP 6848	2016 Jun 21	*Miniopterus natalensis*	Morphologic	Limpopo	–24.11487, 30.12151	Matlapitsi Cave
UP 7094, TM50574	2016 Sep 05	*Miniopterus natalensis*	Museum voucher	Limpopo	–24.6181, 27.65231	Madimatle Cave
UP 7109; NHCPHE MAM-18	2016 Sep 06	*Rhinolophus simulator*	Museum voucher	Limpopo	–24.6181, 27.65231	Madivirology Sammatle Cave
UP 7161	2016 Sep 14	*Miniopterus natalensis*	Morphologic	North West	–26.036079, 26.704976	Die Coetzee Boerdery
UP 7255, TM 50575	2016 Nov 07	*Miniopterus natalensis*	Morphologic	Limpopo	–24.6181, 27.65231	Madimatle Cave
UP 7278; NHCPHE MAM-19	2016 Nov 08	*Rhinolophus simulator*	Museum voucher	Limpopo	–24.6181, 27.65231	Madimatle Cave

Laboratory no.*	Collection date	Bat species†	ID method	Province	Coordinates	SIte
UP 7301, TM50646	2016 Nov 14	*Miniopterus natalensis*	Genetic, Museum voucher	Limpopo	–24.11487, 30.12151	Matlapitsi Cave
UP 7408	2017 Feb 16	*Miniopterus natalensis*	Morphologic	Gauteng	–25.897071, 28.221455	Grootboom Cave, Irene
UP7560	2017 Mar 28	*Rhinolophus clivosus*	Morphologic	Limpopo	–24.6181, 27.65231	Madimatle Cave
UP 7561, TM50583	2017 Mar 28	*Rhinolophus blasii*	Museum voucher	Limpopo	–24.6181, 27.65231	Madimatle Cave
UP 7585, TM50584	2017 Mar 29	*Miniopterus natalensis*	Museum voucher	Limpopo	–24.6181, 27.65231	Madimatle Cave
UP 7766, TM50647	2017 May 03	*Miniopterus natalensis*	Museum voucher	Limpopo	–24.11487, 30.12151	Matlapitsi Cave
UP 7767, TM50648	2017 May 03	*Miniopterus natalensis*	Genetic, Museum voucher	Limpopo	–24.11487, 30.12151	Matlapitsi Cave
UP 7770, TM50650	2017 May 03	*Miniopterus natalensis*	Museum voucher	Limpopo	–24.11487, 30.12151	Matlapitsi Cave
UP 7771, TM50651	2017 May 03	*Miniopterus natalensis*	Museum voucher	Limpopo	–24.11487, 30.12151	Matlapitsi Cave
UP 7880, NHCPHE MAM-69	2017 May 15	*Miniopterus natalensis*	Museum voucher	Limpopo	–24.11487, 30.12151	Matlapitsi Cave
UP 7886, NHCPHE MAM-70	2017 May 15	*Rhinolophus blasii*	Museum voucher	Limpopo	–24.11487, 30.12151	Matlapitsi Cave
UP 7905, NHCPHE MAM-71	2017 May 17	*Nycteris thebaica*	Museum voucher	Limpopo	–24.6181, 27.65231	Madimatle Cave
UP 7922, NHCPHE MAM-62	2017 May 24	*Miniopterus natalensis*	Museum voucher	Gauteng	–25.897071, 28.221455	Grootboom Cave, Irene
UP 7933	2013 Jul 08	*Myotis tricolor*	Morphologic	Limpopo	–24.11487, 30.12151	Matlapitsi Cave
UP 7936	2013 Jul 08	*Myotis tricolor*	Morphologic	Limpopo	–24.11487, 30.12151	Matlapitsi Cave
UP 7937	2013 Sep 03	*Rhinolophus clivosus*	Genetic	Limpopo	–24.11487, 30.12151	Matlapitsi Cave
UP 7943, TM50644	2017 Aug 28	*Nycteris thebaica*	Museum voucher	Limpopo	–24.6181, 27.65231	Madimatle Cave
UP 7949, TM50639	2017 Aug 28	*Miniopterus natalensis*	Museum voucher	Limpopo	–24.6181, 27.65231	Madimatle Cave
UP 7982, TM50655	2017 Sep 07	*Miniopterus natalensis*	Museum voucher	Gauteng	–25.897071, 28.221455	Grootboom Cave, Irene
UP 7983, TM50656	2017 Sep 07	*Miniopterus natalensis*	Museum voucher	Gauteng	–25.897071, 28.221455	Grootboom Cave, Irene
UP 8009	2017 Sep 26	*Rhinolophus simulator*	Morphologic	Limpopo	–24.11487, 30.12151	Matlapitsi Cave
UP 8054	2017 Sep 27	*Rhinolophus simulator*	Morphologic	Limpopo	–24.11487, 30.12151	Matlapitsi Cave
UP 8106	2017 Sep 27	*Miniopterus natalensis*	Genetic	Limpopo	–24.11487, 30.12151	Matlapitsi Cave
UP 8107	2017 Sep 27	*Miniopterus natalensis*	Genetic	Limpopo	–24.11487, 30.12151	Matlapitsi Cave
UP 8132	2017 Sep 28	*Miniopterus natalensis*	Morphologic	Limpopo	–24.11487, 30.12151	Matlapitsi Cave
UP 8162, TM50661	2017 Oct 16	*Miniopterus natalensis*	Museum voucher	Limpopo	–24.11487, 30.12151	Matlapitsi Cave
UP 8167, TM50660	2017 Oct 16	*Miniopterus fraterculus*	Genetic, Museum voucher	Limpopo	–24.11487, 30.12151	Matlapitsi Cave
UP 8207, TM50654	2017 Oct 17	*Rhinolophus simulator*	Museum voucher	Limpopo	–24.11487, 30.12151	Matlapitsi Cave
UP 8235	2017 Oct 17	*Rhinolophus simulator*	Morphologic	Limpopo	–24.11487, 30.12151	Matlapitsi Cave
UP 8356	2017 Nov 07	*Miniopterus natalensis*	Morphologic	Limpopo	–24.11487, 30.12151	Matlapitsi Cave

Laboratory no.*	Collection date	Bat species†	ID method	Province	Coordinates	SIte
UP 8368	2017 Nov 07	*Rhinolophus simulator*	Morphologic	Limpopo	–24.11487, 30.12151	Matlapitsi Cave
UP 8369	2017 Nov 07	*Rhinolophus clivosus*	Morphologic	Limpopo	–24.11487, 30.12151	Matlapitsi Cave
UP 8390	2017 Nov 07	*Miniopterus natalensis*	Genetic	Limpopo	–24.11487, 30.12151	Matlapitsi Cave
UP 8437	2017 Nov 08	*Miniopterus natalensis*	Genetic	Limpopo	–24.11487, 30.12151	Matlapitsi Cave
UP 8485, TM50577	2017 Nov 20	*Nycteris thebaica*	Museum voucher	Limpopo	–24.6181, 27.65231	Madimatle Cave
UP 8488, TM50578	2017 Nov 20	*Miniopterus natalensis*	Museum voucher	Limpopo	–24.6181, 27.65231	Madimatle Cave
UP 8502, TM50579	2017 Nov 21	*Miniopterus natalensis*	Museum voucher	Limpopo	–24.6181, 27.65231	Madimatle Cave
UP 8540, TM50580	2017 Nov 23	*Miniopterus natalensis*	Museum voucher	Limpopo	–24.61335, 27.65381	Randstephne 455
UP 8555, TM50581	2017 Nov 23	*Miniopterus natalensis*	Museum voucher	Limpopo	–24.61335, 27.65381	Randstephne 455
UP 8572, TM50657	2017 Dec 05	*Rhinolophus blasii*	Museum voucher	Limpopo	–24.11487, 30.12151	Matlapitsi Cave
UP 8599	2016 Dec 01	*Pipistrellus rusticus*	Morphologic	Gauteng	–25.8852, 28.3608	Grootfontein Country Estate
UP 8602	2018 Jan 24	*Myotis tricolor*	Morphologic	Limpopo	–24.11487, 30.12151	Matlapitsi Cave
UP 8614	2018 Jan 24	*Rhinolophus clivosus*	Morphologic	Limpopo	–24.11487, 30.12151	Matlapitsi Cave
UP 8615	2018 Jan 24	*Rhinolophus simulator*	Morphologic	Limpopo	–24.11487, 30.12151	Matlapitsi Cave
UP 8667	2018 Feb 07	*Rhinolophus simulator*	Morphologic	Limpopo	–24.11487, 30.12151	Matlapitsi Cave
UP 8668	2018 Feb 07	*Rhinolophus simulator*	Morphologic	Limpopo	–24.11487, 30.12151	Matlapitsi Cave
UP 8676	2018 Feb 07	*Rhinolophus simulator*	Morphologic	Limpopo	–24.11487, 30.12151	Matlapitsi Cave
UP 8679	2018 Feb 07	*Rhinolophus clivosus*	Morphologic	Limpopo	–24.11487, 30.12151	Matlapitsi Cave
UP 8720, TM50604	2018 Feb 26	*Rhinolophus blasii*	Museum voucher	Limpopo	–24.6181, 27.65231	Madimatle Cave
UP 8747, NHCPHE MAM-63	2018 Feb 27	*Neoromicia (Laephotis) capensis*	Museum voucher	Limpopo	–24.11487, 30.12151	Matlapitsi Cave
UP 8748, TM50605	2018 Feb 27	*Rhinolophus simulator*	Museum voucher	Limpopo	–24.6181, 27.65231	Madimatle Cave
UP 8752, NHCPHE MAM-64	2018 Feb 27	*Nycteris thebaica*	Museum voucher	Limpopo	–24.6181, 27.65231	Madimatle Cave
UP 8816	2018 Mar 06	*Rhinolophus clivosus*	Morphologic	Limpopo	–24.11487, 30.12151	Matlapitsi Cave
UP 8874	2018 Apr 10	*Rhinolophus simulator*	Morphologic	Limpopo	–24.11487, 30.12151	Matlapitsi Cave
UP 8876	2018 Apr 10	*Rhinolophus blasii*	Morphologic	Limpopo	–24.11487, 30.12151	Matlapitsi Cave
UP 8879	2018 Apr 10	*Rhinolophus simulator*	Morphologic	Limpopo	–24.11487, 30.12151	Matlapitsi Cave
UP 8929	2018 Apr 10	*Rhinolophus clivosus*	Morphologic	Limpopo	–24.11487, 30.12151	Matlapitsi Cave
UP 8932	2018 Apr 11	*Rhinolophus simulator*	Morphologic	Limpopo	–24.11487, 30.12151	Matlapitsi Cave
UP 9029, TM50606	2018 May 21	*Rhinolophus blasii*	Museum voucher	Limpopo	–24.6181, 27.65231	Madimatle Cave
UP 9042, NHCPHE MAM-65	2018 May 21	*Rhinolophus smithersi*	Museum voucher	Limpopo	–24.6181, 27.65231	Madimatle Cave
UP 9060, TM50607	2018 May 22	*Rhinolophus simulator*	Museum voucher	Limpopo	–24.6181, 27.65231	Madimatle Cave
UP 9075, TM50608	2018 May 23	*Rhinolophus simulator*	Museum voucher	Limpopo	–24.6181, 27.65231	Madimatle Cave

Laboratory no.*	Collection date	Bat species†	ID method	Province	Coordinates	SIte
UP 9689	2018 Jun 06	*Rhinolophus simulator*	Morphologic	Limpopo	–24.11487, 30.12151	Matlapitsi Cave
UP 9745	2018 Jul 18	*Mops midas*	Morphologic	Limpopo	–24.059627, 31.043614	Phalaborwa
UP 9746	2018 Jul 18	*Mops midas*	Morphologic	Limpopo	–24.059627, 31.043614	Phalaborwa
UP 9777, TM50653	2018 Jul 31	*Miniopterus natalensis*	Museum voucher	Limpopo	–24.11487, 30.12151	Matlapitsi Cave
UP 9896, TM50640	2018 Aug 20	*Rhinolophus simulator*	Museum voucher	Limpopo	–24.6181, 27.65231	Madimatle Cave
UP 9938, TM50641	2018 Aug 21	*Miniopterus natalensis*	Museum voucher	Limpopo	–24.6181, 27.65231	Madimatle Cave
UP 9946, TM50642	2018 Aug 22	*Rhinolophus simulator*	Museum voucher	Limpopo	–24.6181, 27.65231	Madimatle Cave
UP 9947, TM50643	2018 Aug 22	*Rhinolophus simulator*	Museum voucher	Limpopo	–24.6181, 27.65231	Madimatle Cave
UP 9963	2018 Sep 04	*Rhinolophus clivosus*	Morphologic	Limpopo	–24.11487, 30.12151	Matlapitsi Cave
UP 9964	2018 Sep 04	*Rhinolophus blasii*	Morphologic	Limpopo	–24.11487, 30.12151	Matlapitsi Cave
UP 10018	2018 Sep 05	*Rhinolophus simulator*	Morphologic	Limpopo	–24.11487, 30.12151	Matlapitsi Cave
UP 10029, TM50613	2018 Oct 02	*Rhinolophus clivosus*	Museum voucher	Limpopo	–24.11487, 30.12151	Matlapitsi Cave
UP 11502, TM48415	2011 Mar 01	*Neoromicia (Laephotis) capensis*	Genetic, Museum voucher	Gauteng	–25.39905, 28.360783	Kwalata Game Ranch
UP 11503, TM48416	2011 Mar 02	*Pipistrellus rusticus*	Museum voucher	Gauteng	–25.39815, 28.35425	Kwalata Game Ranch
UP 11504, TM48432	2011 May 04	*Neoromicia (Laephotis) capensis*	Genetic, Museum voucher	Gauteng	–25.380267, 28.316867	Kwalata Game Ranch
UP 11505, TM48373	2011 Jan 06	*Neoromicia (Laephotis) capensis*	Genetic, Museum voucher	Gauteng	–25.389917, 28.322633	Kwalata Game Ranch
UP 11506, TM48387	2011 Jan 08	*Scotophilus dinganii*	Genetic, Museum voucher	Gauteng	.25.385033, 28.317483	Kwalata Game Ranch
UP 11507, TM48399	2011 Jan 15	*Neoromicia (Laephotis) capensis*	Genetic, Museum voucher	Gauteng	–25.38985, 28.316483	Kwalata Game Ranch
UP 11508, TM48422	2011 Apr 15	*Neoromicia (Laephotis) capensis*	Genetic, Museum voucher	Gauteng	–25.391733, 28.323133	Kwalata Game Ranch
UP 11509, TM48401	2011 Jan 16	*Neoromicia (Laephotis) capensis*	Genetic, Museum voucher	Gauteng	–25.387783, 28.322767	Kwalata Game Ranch
UP 11510, TM48433	2011 May 20	*Neoromicia (Laephotis) capensis*	Genetic, Museum voucher	Gauteng	–25.379083, 28.317467	Kwalata Game Ranch
UP 11511, TM48409	2011 Feb 23	*Neoromicia (Laephotis) capensis*	Genetic, Museum voucher	Gauteng	–25.41567, 28.312167	Kwalata Game Ranch
UP 11512, TM48421	2011 Mar 27	*Neoromicia (Laephotis) capensis*	Museum voucher	Gauteng	–25.389917, 28.322633	Kwalata Game Ranch
UP 11513, TM48408	2011 Jan 29	*Neoromicia (Laephotis) capensis*	Genetic, Museum voucher	Gauteng	–25.379833, 28.321717	Kwalata Game Ranch
UP 11514, TM48398	2011 Jan 14	*Neoromicia (Laephotis) capensis*	Genetic, Museum voucher	Gauteng	–25.4026, 28.337683	Kwalata Game Ranch
UP 11515, TM48391	2011 Jan 08	*Neoromicia (Laephotis) capensis*	Genetic, Museum voucher	Gauteng	–25.385033, 28.317483	Kwalata Game Ranch
UP 11516, TM48392	2011 Jan 08	*Pipistrellus rusticus*	Museum voucher	Gauteng	–25.385033, 28.317483	Kwalata Game Ranch

Laboratory no.*	Collection date	Bat species†	ID method	Province	Coordinates	SIte
UP 11517, TM48393	2011 Jan 08	*Neoromicia (Laephotis) capensis*	Genetic, Museum voucher	Gauteng	–25.385033, 28.317483	Kwalata Game Ranch
UP 11518, TM48371	2011 Jan 04	*Neoromicia (Laephotis) capensis*	Genetic, Museum voucher	Gauteng	–25.382567, 28.3172	Kwalata Game Ranch
UP 11519, TM48372	2011 Jan 04	*Pipistrellus rusticus*	Museum voucher	Gauteng	–25.382567, 28.3172	Kwalata Game Ranch
UP 11520, TM48361	2010 Dec 15	*Laephotis botswanae*	Genetic, Museum voucher	Gauteng	–25.385133, 28.319967	Kwalata Game Ranch
UP 11521, TM48402	2011 Jan 16	*Neoromicia (Laephotis) capensis*	Genetic, Museum voucher	Gauteng	–25.387783, 28.322767	Kwalata Game Ranch
UP 11522, TM48362	2010 Dec 15	*Neoromicia (Laephotis) capensis*	Genetic, Museum voucher	Gauteng	–25.385133, 28.319967	Kwalata Game Ranch
UP 11523, TM48357	2010 Nov 30	*Neoromicia (Laephotis) capensis*	Genetic, Museum voucher	Gauteng	–25.398, 28.312017	Kwalata Game Ranch
UP 11524, TM48363	2010 Dec 15	*Pipistrellus rusticus*	Museum voucher	Gauteng	–25.385133, 28.319967	Kwalata Game Ranch
UP 11525, TM48364	2010 Dec 15	*Pipistrellus rusticus*	Museum voucher	Gauteng	–25.38533, 28.319967	Kwalata Game Ranch
UP 11526, TM48359	2010 Dec 02	*Neoromicia (Laephotis) capensis*	Genetic, Museum voucher	Gauteng	–25.381767, 28.3159	Kwalata Game Ranch
UP 11527, TM48365	2010 Dec 15	*Neoromicia (Laephotis) capensis*	Genetic, Museum voucher	Gauteng	–25.385133, 28.319967	Kwalata Game Ranch
UP 11528, TM48367	2011 Jan 04	*Neoromicia (Laephotis) capensis*	Genetic, Museum voucher	Gauteng	–25.382567, 28.3172	Kwalata Game Ranch
UP 11529, TM48368	2011 Jan 04	*Scotophilus dinganii*	Genetic, Museum voucher	Gauteng	–25.382567, 28.3172	Kwalata Game Ranch
UP 11530, TM48369	2011 Jan 04	*Pipistrellus rusticus*	Museum voucher	Gauteng	–25.382567, 28.3172	Kwalata Game Ranch
UP 11531, TM48403	2011 Jan 18	*Pipistrellus rusticus*	Museum voucher	Gauteng	–25.389917, 28.322633	Kwalata Game Ranch
UP 11532, TM48404	2011 Jan 18	*Pipistrellus hesperidus*	Museum voucher	Gauteng	–25.382567, 28.3172	Kwalata Game Ranch
UP 11533, TM48389	2011 Jan 08	*Pipistrellus rusticus*	Museum voucher	Gauteng	–25.385033, 28.317483	Kwalata Game Ranch
UP 11534, TM48390	2011 Jan 08	*Pipistrellus rusticus*	Museum voucher	Gauteng	–25.382567, 28.3172	Kwalata Game Ranch
UP 11535, TM48406	2011 Jan 22	*Neoromicia (Laephotis) capensis*	Genetic, Museum voucher	Gauteng	–25.39295, 28.3321	Kwalata Game Ranch
UP 11536, TM48396	2011 Jan 14	*Pipistrellus rusticus*	Museum voucher	Gauteng	–25.4026, 28.337683	Kwalata Game Ranch
UP 11537, TM48407	2011 Jan 22	*Neoromicia (Laephotis) capensis*	Genetic, Museum voucher	Gauteng	–25.39295, 28.3321	Kwalata Game Ranch
UP 11538, TM48397	2011 Jan 14	*Pipistrellus rusticus*	Museum voucher	Gauteng	–25.4026, 28.337683	Kwalata Game Ranch
UP 11539, TM48410	2011 Feb 23	*Pipistrellus rusticus*	Museum voucher	Gauteng	–25.415667, 28.312167	Kwalata Game Ranch
UP 11540, TM48411	2011 Feb 23	*Pipistrellus rusticus*	Museum voucher	Gauteng	–25.415667, 28.312167	Kwalata Game Ranch
UP 11541, TM48413	2011 Feb 23	*Pipistrellus rusticus*	Museum voucher	Gauteng	–25.415667, 28.312167	Kwalata Game Ranch
UP 11542, TM48425	2011 Apr 19	*Tadarida aegyptiaca*	Genetic, Museum voucher	Gauteng	–25.415667, 28.312167	Kwalata Game Ranch
UP 11543, TM48426	2011 Apr 19	*Tadarida aegyptiaca*	Genetic, Museum voucher	Gauteng	–25.415667, 28.312167	Kwalata Game Ranch

Laboratory no.*	Collection date	Bat species†	ID method	Province	Coordinates	SIte
UP 11544, TM48426	2011 Apr 19	*Tadarida aegyptiaca*	Genetic, Museum voucher	Gauteng	–25.415667, 28.312167	Kwalata Game Ranch
UP 11545, TM48426	2010 Dec 02	*Pipistrellus rusticus*	Museum voucher	Gauteng	–25.415667, 28.312167	Kwalata Game Ranch
UP 11546, TM48360	2010 Dec 15	*Pipistrellus rusticus*	Museum voucher	Gauteng	–25.415667, 28.312167	Kwalata Game Ranch
UP 11547, TM48366	2011 Jan 04	*Pipistrellus rusticus*	Museum voucher	Gauteng	–25.415667, 28.312167	Kwalata Game Ranch
UP 11548, TM48370	2011 Jan 04	*Pipistrellus rusticus*	Museum voucher	Gauteng	–25.415667, 28.312167	Kwalata Game Ranch
UP 11549, TM48424	2011 Apr 19	*Tadarida aegyptiaca*	Genetic, Museum voucher	Gauteng	–25.415667, 28.312167	Kwalata Game Ranch
UP 11550, TM48434	2011 May 20	*Neoromicia (Laephotis) capensis*	Genetic, Museum voucher	Gauteng	–25.415667, 28.312167	Kwalata Game Ranch
UP 12814, TM48435	2011 May 29	*Scotophilus dinganii*	Genetic, Museum voucher	Gauteng	–25.172, 28.366267	Kwalata Game Ranch
UP 12815, TM48381	2011 Jan 07	*Nycteris thebaica*	Genetic, Museum voucher	Gauteng	–25.38095, 28.345017	Tamboti Lodge
UP 12816, TM48382	2011 Jan 07	*Nycteris thebaica*	Genetic, Museum voucher	Gauteng	–25.38095, 28.345017	Tamboti Lodge
UP 12817, TM48417	2011 Mar 02	*Neoromicia (Laephotis) capensis*	Genetic, Museum voucher	Gauteng	–25.392950, 28.3321	Kwalata Game Ranch
UP 12818, TM48400	2011 Jan 15	*Neoromicia (Laephotis) capensis*	Genetic, Museum voucher	Gauteng	–25.389850, 28.316483	Kwalata Game Ranch
UP 12819, TM48385	2011 Jan 07	*Nycteris thebaica*	Genetic, Museum voucher	Gauteng	–25.38095, 28.345017	Tamboti Lodge
UP 12820, TM48386	2011 Jan 07	*Nycteris thebaica*	Genetic, Museum voucher	Gauteng	–25.38095, 28.345017	Tamboti Lodge
UP 12821, TM48418	2011 Mar 02	*Neoromicia capensis*	Genetic, Museum voucher	Gauteng	–25.392950, 28.3321	Kwalata Game Ranch
UP 12822, TM48430	2011 Apr 19	*Neoromicia (Laephotis) capensis*	Genetic, Museum voucher	Gauteng	–25.382533, 28.3167	Kwalata Game Ranch
UP 12823, TM48412	2011 Feb 23	*Pipistrellus rusticus*	Museum voucher	Gauteng	–25.415667, 28.312167	Kwalata Game Ranch
UP 12824, TM48395	2011 Jan 14	*Neoromicia (Laephotis) capensis*	Genetic, Museum voucher	Gauteng	–25.402600, 28.337683	Kwalata Game Ranch
UP 12825, TM48405	2011 Jan 18	*Neoromicia (Laephotis) capensis*	Genetic, Museum voucher	Gauteng	–25.389917, 28.322633	Kwalata Game Ranch
UP 12826, TM48374	2011 Jan 07	*Nycteris thebaica*	Genetic, Museum voucher	Gauteng	–25.38095, 28.345017	Tamboti Lodge
UP 12827, TM48419	2011 Mar 13	*Neoromicia (Laephotis) capensis*	Genetic, Museum voucher	Gauteng	–25.402650, 28.3377	Kwalata Game Ranch
UP 12828, TM48436	2011 Jan 21	*Pipistrellus rusticus*	Museum voucher	Gauteng	–25.399933, 28.352517	Kwalata Game Ranch
UP 12829, TM48420	2011 Mar 26	*Pipistrellus rusticus*	Museum voucher	Gauteng	–25.399933, 28.352517	Kwalata Game Ranch
UP 12830, TM48380	2011 Jan 07	*Nycteris thebaica*	Genetic, Museum voucher	Gauteng	–25.38095, 28.345017	Tamboti Lodge
UP 12831, TM48423	2011 Apr 19	*Tadarida aegyptiaca*	Genetic, Museum voucher	Gauteng	–25.417083, 28.31165	Kwalata Game Ranch

Laboratory no.*	Collection date	Bat species†	ID method	Province	Coordinates	SIte
UP 12832, TM48383	2011 Jan 07	*Nycteris thebaica*	Genetic, Museum voucher	Gauteng	–25.38095, 28.345017	Tamboti Lodge
UP 12833, TM48384	2011 Jan 07	*Nycteris thebaica*	Genetic, Museum voucher	Gauteng	–25.38095, 28.345017	Tamboti Lodge
UP 12834, TM48375	2011 Jan 07	*Nycteris thebaica*	Genetic, Museum voucher	Gauteng	–25.38095, 28.345017	Tamboti Lodge
UP 12835, TM48394	2011 Jan 08	*Nycteris thebaica*	Genetic, Museum voucher	Gauteng	–25.385033, 28.317483	Kwalata Game Ranch
UP 12836, TM48427	2011 Apr 19	*Tadarida aegyptiaca*	Genetic, Museum voucher	Gauteng	–25.417083, 28.31165	Kwalata Game Ranch
UP 12837, TM48428	2011 Apr 19	*Tadarida aegyptiaca*	Genetic, Museum voucher	Gauteng	–25.417083, 28.31165	Kwalata Game Ranch
UP 12838, TM48431	2011 Apr 19	*Neoromicia capensis*	Genetic, Museum voucher	Gauteng	–25.382533, 28.3167	Kwalata Game Ranch
UP 12839, TM48376	2011 Jan 07	*Nycteris thebaica*	Genetic, Museum voucher	Gauteng	–25.38095, 28.345017	Tamboti Lodge
UP 12840, TM48377	2011 Jan 07	*Nycteris thebaica*	Genetic, Museum voucher	Gauteng	–25.38095, 28.345017	Tamboti Lodge
UP 12841, TM48378	2011 Jan 07	*Nycteris thebaica*	Genetic, Museum voucher	Gauteng	–25.38095, 28.345017	Tamboti Lodge
UP 12842, TM48414	2011 Feb 23	*Neoromicia (Laephotis) capensis*	Genetic, Museum voucher	Gauteng	–25.415667, 28.312167	Kwalata Game Ranch
UP 12843, TM48379	2011 Jan 07	*Nycteris thebaica*	Genetic, Museum voucher	Gauteng	–25.38095, 28.345017	Tamboti Lodge

*Catalog nos. of voucher specimens are provided after the UP no. DM, Durban Natural Science Museum; NHCPHE MAM, Natural History Collection for Public Health and Economics; TM, Ditsong National Museum of Natural History; UP, University of Pretoria.
†Updated taxonomic names are indicated in parentheses.

Appendix Table 2. Bat species sampled for lyssavirus, South Africa, 2003–2018

Bat species*	Province, no. bats collected						Total, no.
	Eastern Cape	Gauteng	KwaZulu-Natal	Limpopo	Mpumalanga	North West	
Chaerephon (Mops) ansorgei	0	0	0	2	0	0	2
Chaerephon (Mops) pumilus	0	0	15	3	0	0	18
Cloeotis percivali	0	0	0	5	0	0	5
Eptesicus hottentotus	0	0	0	0	0	1	1
Glauconycteris variegate	0	0	3	1	0	0	4
Hipposideros caffer	0	0	0	16	1	0	17
Kerivoula (*Glauconycteris*) *argentata*	0	0	1	0	0	0	1
Laephotis botswanae	0	1	1	0	0	0	2
Miniopterus fraterculus	0	1	0	2	0	2	5
Miniopterus natalensis	0	30	8	80	0	6	124
Mops condylurus	0	1	0	8	0	0	9
Mops midas	0	0	0	3	0	0	3
Myotis tricolor	0	0	0	9	2	0	11
Myotis welwitschii	0	0	0	0	1	0	1
Neoromicia (Laephotis) capensis	0	42	3	7	4	11	67
Neoromicia (Laephotis) cf. helios	0	0	0	4	0	0	4
Neoromicia (Laephotis) helios	0	0	0	2	0	0	2
Neoromicia (Laephotis) nana	0	0	2	8	3	0	13
Neoromicia (Laephotis) rendalli	0	0	0	1	0	0	1
Neoromicia (Laephotis) zuluensis	0	0	0	2	1	0	3
Nycteris thebaica	0	14	9	18	0	1	42
Nycticeinops schlieffeni	0	0	0	8	0	0	8
Otomops martiensseni	0	0	7	0	0	0	7
Pipistrellus hesperidus	7	1	9	2	1	0	20
Pipistrellus rusticus	0	23	0	8	1	3	35
Rhinolophus blasii	0	0	0	12	0	0	12
Rhinolophus clivosus	0	2	10	17	1	0	30
Rhinolophus damarensis	0	0	0	0	0	5	5
Rhinolophus darlingi	0	0	1	4	0	0	5
Rhinolophus denti	0	0	0	0	0	5	5
Rhinolophus hildebrandtii s.l.	0	0	0	1	2	0	3
Rhinolophus landeri	0	0	0	1	0	0	1
Rhinolophus simulator	0	0	0	48	0	2	50
Rhinolophus smithersi	0	0	0	4	0	0	4
Rhinolophus swinnyi	0	0	3	0	0	0	3
Sauromys petrophilus	0	0	0	0	0	1	1
Scotophilus dinganii	0	5	14	18	0	12	49
Scotophilus leucogaster	0	0	0	5	0	0	5
Scotophilus viridis	0	0	0	7	0	0	7
Tadarida aegyptiaca	0	10	2	0	0	5	17
Taphozous mauritianus	0	0	0	3	0	0	3
Total	7	130	88	309	17	54	605

*Updated taxonomic names are indicated in parentheses

Appendix Table 3. Provincial permits to conduct lyssavirus surveillance among insectivorous bats, South Africa, 2003–2018

Issuer	Permit no.	Permit title	Date issued
Limpopo Provincial Government, Department of Economic Development, Environment and Tourism	A02–005–00003	Collect mammals for scientific purposes	2004 Feb 16
	CPM-005–00010	Collect mammals for scientific purposes	2007 Mar 30
	RB/2010/22	Permit to move animals	2010 Oct 15
	001-CPM402–0007	Scientific research on mammals	2013 Feb 08
	ZA/LP/84188	Scientific research on mammals	2017 Aug 29
	ZA/LP/83642	Scientific research on mammals	2017 Aug 03
	CPM006806	Scientific research on mammals	2012 May 03
	ZA/LP/73972	Scientific research on mammals	2016 Feb 05
	ZA/LP/91509	Scientific research on mammals	2018 Sep 06
	ZA/LP92784	Scientific research on mammals	2018 Nov 08
Gauteng Provincial Government, Department of Agriculture, Conservation, Environment	CPF6 No. 0027	Collect and convey wild animals for scientific purposes	2010 Apr 29
	CPF6 No. 0109	Collect and convey wild animals for scientific purposes	2014 Mar 04
	CPB6 No. 003767	Import into province wild animals	2012 Nov 24
	CPF6 No. 0123	Collect and convey wild animals for scientific purposes	2016 Apr 06
KwaZulu-Natal Wildlife Permits Office	OP 2098/2015	Mammal capture and export	2015 May 18
North-West Province, Department of Agriculture, Conservation Environment and Tourism	000039 NW-07	Collection of bats for scientific purposes	2007 Feb 19
Mpumalanga Tourism and Parks Agency	MPB5177	Catch/collect and convey mammals for scientific purposes	2007 Jan 05
	MPB5385	Catch/collect and convey mammals for scientific purposes	2014 Feb 10

Appendix Table 4. Selected sequences of lyssaviruses used for phylogenetic analysis*

Genbank accession no.	Virus	Host	Year	Country
EU293108	Lagos bat virus	*Eidolon helvum*	1985	Senegal
EU293110	Lagos bat virus	*Eidolon helvum*	1956	Nigeria
GU170202	Lagos bat virus	*Rousettus aegyptiacus*	2008	Kenya
DQ499944	Lagos bat virus	*Epomophorus wahlbergi*	2003	South Africa
DQ499945	Lagos bat virus	*Epomophorus wahlbergi*	2004	South Africa
DQ499946	Lagos bat virus	*Epomophorus wahlbergi*	1980	South Africa
DQ499947	Lagos bat virus	*Epomophorus wahlbergi*	1982	South Africa
EF547447	Lagos bat virus	*Rousettus aegyptiacus*	1999	France (imported)
EF547449	Lagos bat virus	*Micropterus pusillus*	1974	Central African Republic
EF547450	Lagos bat virus	*Felis catus*	1986	Zimbabwe
EF547452	Lagos bat virus	*Epomophorus wahlbergi*	2006	South Africa
EU259198	Lagos bat virus	*Eidolon helvum*	2007	Kenya
HM179509	Lagos bat virus	*Epomophorus wahlbergi*	2008	South Africa
JX901139	Lagos bat virus	*Rousettus aegyptiacus*	2010	Kenya
LN849915	Lagos bat virus	*Eidolon helvum*	2013	Ghana
AF418014	Australian bat lyssavirus	*Homo sapiens*	1998	Australia
KT868956	Australian bat lyssavirus	*Pteropus scapulatus*	2014	Australia
NC003243	Australian bat lyssavirus	*Saccolaimus flaviventris*	1996	Australia
KT868955	Australian bat lyssavirus	*Pteropus alecto*	2014	Australia
AY573937	Australian bat lyssavirus	*Saccolaimus flaviventris*	1997	Australia
AY573963	Australian bat lyssavirus	*Pteropus alecto*	1998	Australia
AY573964	Australian bat lyssavirus	*Pteropus alecto*	1997	Australia
AY573965	Australian bat lyssavirus	*Saccolaimus flaviventris*	1997	Australia
KF155004	European bat lyssavirus 2	*Myotis daubentonii*	2004	United Kingdom
KY688152	European bat lyssavirus 2	*Myotis dasycneme*	1993	Netherlands
NC009528	European bat lyssavirus 2	*Homo sapiens*	2002	United Kingdom
EU293114	European bat lyssavirus 2	*Myotis dasycneme*	1986	Netherlands
AY863364	European bat lyssavirus 1	*Pteropus alecto*	2000	Netherlands

Genbank accession no.	Virus	Host	Year	Country
AY863382	European bat lyssavirus 1	*Eptesicus serotinus*	2001	Slovakia
AY863397	European bat lyssavirus 1	*Eptesicus serotinus*	2000	France
AY863405	European bat lyssavirus 2	*Myotis dasycneme*	1989	Netherlands
AY863407	European bat lyssavirus 2	*Myotis daubentonii*	1993	Switzerland
AY863408	European bat lyssavirus 2	NA	2002	Switzerland
EU293119	Duvenhage virus	*Homo sapiens*	1971	South Africa
EU293120	Duvenhage virus	*Miniopterus sp*	1981	South Africa
JN986749	Duvenhage virus	*Homo sapiens*	2007	Netherlands (imported from Kenya)
EU623444	Duvenhage virus	*Homo sapiens*	2006	South Africa
EU626552	European bat lyssavirus 1	*Felis catus*	2007	France
KP241939	European bat lyssavirus 1	*Eptesicus isabellinus*	2007	Spain
NC009527	European bat lyssavirus 1	*Eptesicus serotinus*	1968	Germany
KF155003	European bat lyssavirus 1	*Eptesicus serotinus*	1986	Denmark
EF157976	European bat lyssavirus 1	*Eptesicus serotinus*	1968	Germany
KF977826	Rabies virus	*Homo sapiens*	2011	Central African Republic
KM594028	Rabies virus	*Eptesicus furinalis*	2010	Brazil
KP723638	Rabies virus	*Canis simensis*	2014	Ethiopia
KT336437	Rabies virus	*Canis lupus familiaris*	2012	South Africa
AF351847	Rabies virus	*Desmodus rotundus*	1986	Brazil
AF351850	Rabies virus	Insectivorous bat	1988	Chile
AF374721	Rabies virus	*Canis familiaris*	NA	India
AY352493	Rabies virus	*Homo sapiens*	NA	India
GU358653	Rabies virus	*Canis familiaris*	1994	China
HQ450386	Rabies virus	*Canis familiaris*	NA	Mexico
MF043188	Bokeloh bat lyssavirus	*Myotis nattereri*	2013	France
KC169985	Bokeloh bat lyssavirus	*Myotis nattereri*	2012	France
MF197740	Bokeloh bat lyssavirus	*Myotis nattereri*	2016	Poland
MF472709	Taiwan bat lyssavirus	*Pipistrellus abramus*	2017	Taiwan
MF472710	Taiwan bat lyssavirus	*Pipistrellus abramus*	2016	Taiwan
NC018629	Ikoma lyssavirus	*Civettictis civetta*	2009	Tanzania
NC020808	Aravan virus	*Myotis blythi*	1991	Kyrgyzstan
NC020809	Irkut virus	*Murina leucogaster*	2002	Russia
NC025365	Shimoni bat virus	*Macronycteris vittatus*	2009	Kenya
NC025377	West Caucasian bat virus	*Miniopterus schreibersii*	2002	Russia
NC025385	Khujand virus	*Myotis mystacinus*	2001	Tajikistan
NC031955	Lleida bat lyssavirus	*Miniopterus schreibersii*	2011	Spain
NC031988	Gannoruwa bat lyssavirus	*Pteropus giganteus*	2015	Sri Lanka
KC866301	Duvenhage virus	*Nycteris thebaica*	2012	South Africa
MT364249	Matlo bat lyssavirus	*Miniopterus natalensis*	2015	South Africa
MT364250	Matlo bat lyssavirus	*Miniopterus natalensis*	2016	South Africa
KF155005	Mokola virus	*Crocidura sp.*	1968	Nigeria
EU293117	Mokola virus	*Crocidura sp.*	1974	Cameroon
KF155006	Mokola virus	*Felis catus*	1982	Zimbabwe
KF155007	Mokola virus	*Felis catus*	1993	Zimbabwe
KF155008	Mokola virus	*Felis catus*	1996	South Africa
EU293118	Mokola virus	*Laphuromys sikapusi*	1981	Central African Republic
FJ465410	Mokola virus	*Felis catus*	1998	South Africa
FJ465412	Mokola virus	*Felis catus*	2006	South Africa
FJ465413	Mokola virus	*Felis catus*	1997	South Africa
FJ465414	Mokola virus	*Felis catus*	1996	South Africa
FJ465415	Mokola virus	*Felis catus*	1995	South Africa
FJ465417	Mokola virus	*Felis catus*	1981	Zimbabwe
FJ465418	Mokola virus	*Felis catus*	1981	Zimbabwe
JN944637	Mokola virus	*Felis catus*	1997	South Africa
KC218932	Mokola virus	*Felis catus*	1981	Zimbabwe
KC218934	Mokola virus	*Felis catus*	2008	South Africa
KP899610	Mokola virus	*Felis catus*	2012	South Africa
KP899611	Mokola virus	*Felis catus*	2012	South Africa
KP899612	Mokola virus	*Felis catus*	2014	South Africa
MF960865	Kotalahti bat lyssavirus	*Myotis brandtii*	2017	Finland

*NA, not available.
